# Dispersal syndromes drive the formation of biogeographical regions, illustrated by the case of Wallace’s Line

**DOI:** 10.1111/geb.13250

**Published:** 2021-01-06

**Authors:** Alexander E. White, Kushal K. Dey, Matthew Stephens, Trevor D. Price

**Affiliations:** ^1^ Office of the Chief Information Officer Smithsonian Institution Washington DC USA; ^2^ Department of Botany National Museum of Natural History Smithsonian Institution Washington DC USA; ^3^ Department of Ecology and Evolution University of Chicago Chicago IL USA; ^4^ Department of Epidemiology Harvard T. H. Chan School of Public Health Boston MA USA; ^5^ Department of Statistics University of Chicago Chicago IL USA

**Keywords:** biogeographical regionalization, biotas, birds, dispersal, grade of membership model, Indo‐Pacific, mammals, species assemblages, Sulawesi, Wallace’s Line

## Abstract

**Aim:**

Biogeographical regions (realms) reflect patterns of co‐distributed species (biotas) across space. Their boundaries are set by dispersal barriers and difficulties of establishment in new locations. We extend new methods to assess these two contributions by quantifying the degree to which realms intergrade across geographical space and the contributions of individual species to the delineation of those realms. As our example, we focus on Wallace’s Line, the most enigmatic partitioning of the world’s faunas, where climate is thought to have little effect and the majority of dispersal barriers are short water gaps.

**Location:**

Indo‐Pacific.

**Time period:**

Present day.

**Major taxa studied:**

Birds and mammals.

**Methods:**

Terrestrial bird and mammal assemblages were established in 1‐degree map cells using range maps. Assemblage structure was modelled using latent Dirichlet allocation, a continuous clustering method that simultaneously establishes the likely partitioning of species into biotas and the contribution of biotas to each map cell. Phylogenetic trees were used to assess the contribution of deep historical processes. Spatial segregation between biotas was evaluated across time and space in comparison with numerous hard realm boundaries drawn by various workers.

**Results:**

We demonstrate that the strong turnover between biotas coincides with the north‐western extent of the region not connected to the mainland during the Pleistocene, although the Philippines contains mixed contributions. At deeper taxonomic levels, Sulawesi and the Philippines shift to primarily Asian affinities, resulting from transgressions of a few Asian‐derived lineages across the line. The partitioning of biotas sometimes produces fragmented regions that reflect habitat. Differences in partitions between birds and mammals reflect differences in dispersal ability.

**Main conclusions:**

Permanent water barriers have selected for a dispersive archipelago fauna, excluded by an incumbent continental fauna on the Sunda shelf. Deep history, such as plate movements, is relatively unimportant in setting boundaries. The analysis implies a temporally dynamic interaction between a species’ intrinsic dispersal ability, physiographic barriers, and recent climate change in the genesis of Earth’s biotas.

## 1 INTRODUCTION

Terrestrial biodiversity is structured according to geographical ranges that are shared among taxa. Historically, the geographical extents of associated species have been delimited as realms (Holt et al., 2013; Wallace, 1876), defined as the spatial representation of co‐occurrence patterns among groups of species, termed biotas. While biotas and realms are conceptually separable, they have been difficult to disentangle (Vilhena & Antonelli, 2015), with the consequence that realms have been treated as geographically cohesive and where two realms meet they are placed as sharply abutting with a discrete boundary. Turnover between such realms has been attributed to climate, as exemplified by the tropical–temperate transition (White et al., 2019), and dispersal obstacles such as large water gaps or mountain valleys (Antonelli, 2017; Ficetola et al., 2017; Hazzi et al., 2018). The structure of abutting realms may be mediated by limits to dispersal, which include ease of movement of individuals over barriers, limits imposed by conditions in recipient areas, including climate and incumbent biotas, and, in deep time, the movement of continents. Dispersal dynamics imply that realms should intergrade (Vilhena & Antonelli, 2015) with different biotas intermixing along a geographical zone of transition. In addition, biotas need not be geographically cohesive. They could, for example, occupy patches of distinctive habitat across a region that are readily colonized by a group of species.

**FIGURE 1:**
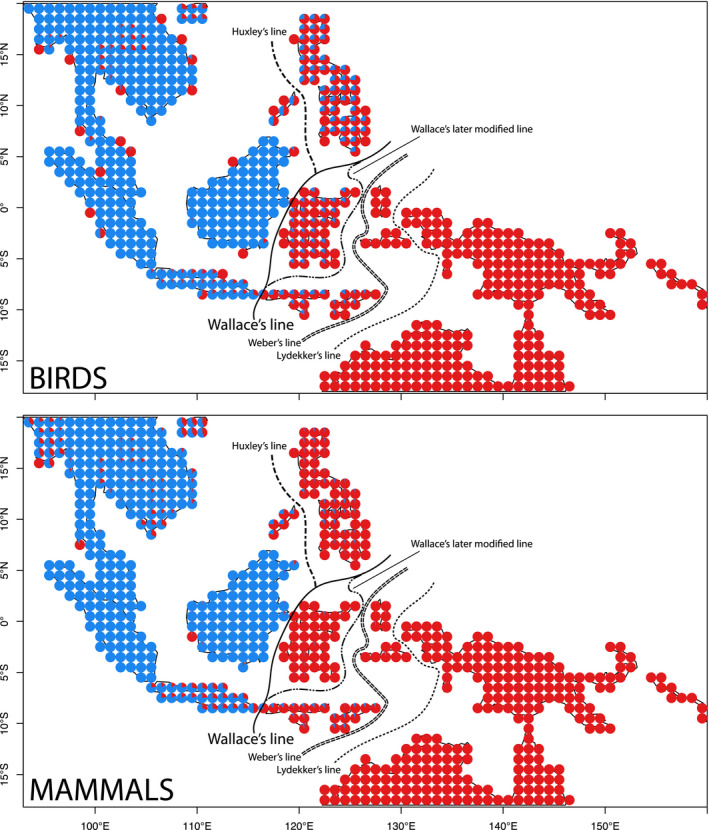
Species motifs for birds and mammals in the Indo‐Pacific, *K* = 2. Each pie chart is coloured according to contributions to the location from the two regional biotas. Birds and mammals were analysed separately (colours represent unique groups in each map). Wallace’s original line is shown along with Huxley’s modification and Wallace’s own later modification. Weber’s and Lydekker’s lines are shown south‐east of Wallace’s Line. Lines are redrawn from van Welzen et al. (2011)

A documentation of the way biotas populate realms is required to understand how and why regions are composed of unique sets of species, which surely represent a combination of historical and present‐day factors limiting dispersal. Wallace’s Line, where Asian and Australian faunas famously meet, is the most enigmatic of all realm boundaries because quite discrete faunas and floras are separated by a short water gap (van Oosterzee, 1997) across which climatic turnover is relatively weak. Major realm boundaries that separate continental biotas reflect climatically disparate geographical regions, such as the line of regular freezing (White et al., 2019). By contrast, only 35 km separates climatically similar Lombok from Bali, which lie either side of Wallace’s original line (Figure 1), suggesting a strong role for historical processes affecting dispersal, rather than present‐day climate. The causes of Wallace’s Line have been extensively studied for more than 100 years (Simpson, 1977) and continue to be active areas of research (Ali et al., 2020; Wainwright et al., 2018).

Investigations centre on what species cross the line, as well as where it lies. Although turnover in the general region of the Wallace’s Line is clear, many species have ranges extending into the islands of Wallacea (Dickerson, 1928), originally defined as the island region between Huxley’s and Lydekker’s lines (Figure 1). Consequently, biologists have long recognized that the ‘line is not without width’ (Pelseneer, 1904; see also Esselstyn et al., 2010; Mayr, 1944). Despite this, methods to date have of necessity assigned sharp boundaries to realms (Holt et al., 2013; Simpson, 1977). The need to draw lines without width led to an active debate among early biogeographers about where the line should be drawn. Seven early biogeographers proposed some modification of Wallace’s original boundary [see Simpson (1977) for a detailed review of each line in turn]. Wallace himself grouped Sulawesi with an Australian realm in his earlier writings, but later with the Asian realm, where it has also been placed in more recent quantitative treatments (Holt et al., 2013). Other lines include Huxley’s modification of Wallace’s original line (Huxley, 1868), Weber’s (Mayr, 1944; Pelseneer, 1904) and Lydekker’s lines (Lydekker, 1896, Figure 1). The first botanical study considered yet a different line, uniting east Java with Australia (van Welzen et al., 2011; island names are given in Supporting Information Figure S1). In summary, the dispute has been not only on how sharp the turnover is, but also where the turnover takes place. This limits our ability to assess the processes that have led to the delineation of realms in the region and the putative factors driving the formation of realms more broadly.

Vilhena and Antonelli (2015) recognized these conceptual issues. They used a network approach to identify biogeographical zones of transition, but rather than directly quantifying the mixing of biotas along a realm boundary, the method assigns transition zones (i.e., the region of biotic mixing across the realm boundary) to discrete clusters of their own. For example, workers in the Indo‐Pacific have long debated whether Wallacea should be defined as a biogeographical unit on its own or if these island assemblages merely represented mixed biotas from the two larger adjacent realms. With discrete clustering methods, the placement of Wallacea as a unique biogeographical entity remains an open question, hindering our understanding of how biotic mixing contributes to the mechanisms of realm delineation.

Here, we leverage a technique for identifying biotas and associated realms using continuous clustering. This approach allows a quantitative interpretation of not only the structure of the transition zone itself, but also, in turn, how the transition may have formed in the face of the various influences of dispersal (Valle et al., 2014; White et al., 2019). We generate the biotas and quantify their spatial extents using a probabilistic model that finds the most likely lower dimensional factorization of the data set into groups, which we term motifs (here equivalent to biotas), and the likely proportions of mixing of those motifs in each location (White et al., 2019). We use pie charts to show the intergradation of the contributions of motifs to each location as a mixture of colours. The model is similar to that applied in population genetics to evaluate the mixing of genetic ancestry within individuals (ADMIXTURE, Pritchard et al., 2000) and models used in natural language processing (Blei, 2012; Blei et al., 2003). Generally, these models are referred to as grade of membership models (Erosheva & Fienberg, 2005).

We use two publicly available data sets describing the geographical distributions of all known birds (BirdLife International NatureServe, 2014) and mammals (IUCN, 2017) together with molecular phylogenies of both groups (Jetz et al., 2012; Upham et al., 2019) to assess the biotic mixing of species across the region encompassing Wallace’s Line (from northern Australia to the southern tip of mainland Asia). We fit our model to generate species motifs for different values of partitioning in both birds and mammals. By comparing analyses of birds and mammals, we show how flight plays a role in generating geographically disjunct biotas across the region as well as promoting transgressions of species across realm boundaries. We then time slice the phylogenies to study the distributions of deep lineages, allowing an investigation of how these patterns are recast when accounting for various levels of relatedness among modern taxa and, in turn, the potential effects of historical movements of lineages across the region in generating biotas. Finding that large islands (e.g. Philippines or Sulawesi) swap Asian or Australian affinities in the lineage‐based analysis shows how movements of taxa in the recent past can generate biotic patterns that differ from those based on clades.

We use the emergent patterns to build up an explanation, based on dispersal and limits to dispersal, that defines the distribution and intergradation of biotas across the region. In particular, we discuss the extent to which the two primary components of range expansions – geographical limits on movement and ecological limits on population establishment (Borregard et al., 2016; White, 2016) – serve as proximate mechanisms for the biogeographical regionalization of the Indo‐Pacific.

## 2 MATERIALS AND METHODS

### 2.1 Species presences

We overlaid global breeding range distributions for all birds and mammals on a 1° × 1° raster grid encompassing southern Thailand, Malaysia, Borneo, Indonesia, the Philippines, Papua New Guinea and northern Australia (longitudinal and latitudinal limits: 90° E, 160° E, 18° S, 20° N; a map with place names can be found in Supporting Information Figure S1). Bird ranges were obtained from BirdLife International and NatureServe (2014) (Version 7, http://datazone.birdlife.org), downloaded on 17 April 2015. Mammal ranges were obtained from the International Union for Conservation of Nature (IUCN; Version 3, May 2017, http://www.iucnredlist.org), downloaded on 10 March 2018. We counted each species as present in a raster cell if the species’ breeding range polygon overlapped the cell. We only counted cells that were composed of more than approximately 1/16 land (estimated by setting *precision* = 0.25 in the *dsp_create_from_gdb()* function in the R package *ecostructure*; White et al., 2019, https://kkdey.github.io/ecostructure). This removed a number of islands of small area and low species diversity from our analysis – indeed any assessment of co‐occurrence patterns (and the underlying co‐diversity, i.e. compositional patterns of sites) is contingent on the number of cells in the analysis and a comparison of the proportional range sizes of species in those cells (Arita, 2017). We reduced our analysis to larger landmasses so as to not bias our clustering towards species associated with small islands, and therefore, potentially widespread, distributions. Although this approach has the potential to exclude locations harbouring small island endemics, we are focused on broad biogeographical patterns that would not likely be meaningfully impacted by species that are only present at a single site. The resulting matrices comprised 2,301 bird species in 664 map cells, and 1,160 mammal species in those same cells. Manipulation of species distributions was done using *ecostructure*, which provides functionality for generating presence–absence matrices from GIS data using the *fasterize* (Ross, 2018), *raster* (Hijmans, 2019) and *sf* (Pebesma, 2018) packages.

### 2.2 Estimating biotas in the Indo‐Australian Archipelago

We estimate biotas using presence–absence matrices of birds and mammals separately. We applied a Bernoulli version of the grade of membership model on each presence–absence data matrix *M_N×G_ =* ((*m_ng_*)) where *m_ng_* is 0/1 based on if the species *g* is absent/present in map cell *n*.

(1) $$m_{\mathrm{ng}}∼Ber\left(p_{\mathrm{ng}}\right)$$

where *p_ng_* is the probability that species *g* is present in the map cell *n*. We assume a lower dimensional representation for *p_ng_*.

(2) $$p_{\mathrm{ng}}=\sum_{k=1}^{K}\omega_{\mathrm{nk}}\theta_{\mathrm{kg}}$$

Where

(3) $$\begin{array}{c} 0\leq \omega_{\mathrm{nk}}\leq 1\;\;\;\;\;\;\;\;\;\sum_{k=1}^{K}\omega_{\mathrm{nk}}=1\;\;\;\;\;\;\;\;\;\;\forall n\\ 0\leq \theta_{\mathrm{kg}}\leq 1\;\;\;\;\;\;\;\;\;\forall k\forall g \end{array}$$

Here, *K* represents the number of underlying motifs fitted in the model, ω*_nk_* represents the proportional contribution of the *k* ^th^ motif to map cell *n* and θ*_kg_* is probability of observing the *g* ^th^ species at a location that belongs (entirely) to the *k* ^th^ motif. We assume Dirichlet priors on the proportions vector ω*_n_*_._ and beta priors for each θ*_kg_* (White et al., 2019).

We fit this model for different values of *K* ranging from 2 to 10. For each value of *K*, we report the best fitted model across 10,000 runs with different initializations, where model fit is assessed in terms of Bayesian information criterion (BIC) or Bayes factors (Valle et al., 2014; White et al., 2019). The membership proportions vector ω*_n_*_._ for each map cell *n* is displayed using a pie chart, placed at the latitude and longitude of map cell *n*. At a 1° × 1° resolution, this visualization shows both the spatial distribution of the motifs and, crucially, the transition between motifs across space. The θ*_k_*_._ vector of probabilities represents the probability that a given species is a member of species motif *k*. We used the function *ExtractTopFeatures* in *ecostructure* to identify the species that uniquely contribute to a single species motif.

### 2.3 Assessing phylogenetic history

We obtained phylogenetic trees for all birds (Jetz et al., 2012) and mammals (Upham et al., 2019) to assess imprints of history on the motifs. We trimmed each phylogeny to include only the species present in the region of interest, but due to the absence of phylogenetic data for a subset of species in the spatial data sets, we were left with 2,167 bird species and 1,133 mammal species for this portion of the analysis. We first assessed phylogenetic differences, essentially phylogenetic beta diversity (Graham & Fine, 2008), between the Asian and Australian biotas using the output from the model fits for *K* = 2, which we show separates realms across Wallace’s Line. Using the θ*_k_*_._ vector, we generated relative θ*_kg_* values per species by dividing θ*_kg_* values for each motif by the summed θ*_kg_* values across the two motifs. We refer to these relative θ*_kg_* values as η*_kg_*. We then pruned the phylogeny to those species with η*_kg_* values entirely falling into one motif, for example, in one motif η*_kg_* = 1, and in the other η*_kg_* = 0. We calculated the Bray–Curtis dissimilarity between the species assigned to each motif as a measure of beta diversity (Baselga, 2013), weighting the contribution of each species to the motifs using the θ*_kg_* values. To compare ages of the two biotas, we computed Faith’s phylogenetic diversity (summed branch lengths, Faith, 1992) from pruned phylogenies of these species, using a standardized phylogenetic diversity (PD) metric (Tsirogiannis & Sandel, 2015) that accounts for the differing number of species in each group. Finally, we estimated phylogenetic beta diversity between motifs (Graham & Fine, 2008) using the UniFrac measure (Lozupone et al., 2007). We used the R packages *betapart* (for Bray–Curtis dissimilarities; Baselga & Orme, 2012) and *PhyloMeasures* (for standardized PD and UniFrac metrics; Tsirogiannis & Sandel, 2015).

Species motifs are based on species distributions, so that, for example, if two sisters are island endemics they would contribute little to the formation of motifs at *K* = 2, given the large area each motif necessarily covers (at *K* = 10, co‐distributed endemic species become more relevant, and as we show, large islands then become zoogeographical regions). Hence, we generated phylogenetic motifs (White et al., 2019) to examine how spatial patterns of partitioning are upheld when species are grouped into clades of a given age. This grouping (a) increases areal extent of a lineage, (b) removes any taxonomic artefacts, and (c) typically links together ecologically similar forms. To generate a phylogenetic motif, we cut the phylogenetic trees at a certain time slice, *T*, at which point some lineages in the tree would subtend one or more related species. We obtained a binary presence/absence at each location for each tip present in the phylogeny at time slice *T* by determining if any of the subtended tips were > 0 in the original presence–absence matrix. In this way, species presences were converted to lineage presences, with the geographical distribution of each lineage representing the union of all the geographical distributions for species descended from that lineage at time *T*. We then fit the grade of membership model for values of *K* = 2…10 to the resulting matrix data matrix with the 664 map cells along the rows and the common ancestors to species at time *T* along the columns.

## 3 RESULTS

### 3.1 The basic partition

The grade of membership model with *K* = 2 captures strong biotic turnover, largely matching Huxley’s modification of Wallace’s original line, placing Sulawesi and the Philippines with the Australian biota (Figure 1; contrary to Huxley, Palawan has a strong Australian affinity). For both birds and mammals, the sharp transition between the islands of Bali and Lombok is apparent (Figure 1). Patterns are similar for birds and mammals, but birds of the Philippines have a stronger mixing with the Asian motif than mammals.

Nearly all of the species were assigned either a 0 or 1 probability (based on η*_kg_*) of belonging to either the Asian or Australian species motif (783 bird species assigned to the Asian motif and 1,196 to the Australian motif, corresponding figures for mammals are 553 and 415, respectively). Many of these are island endemics, but others have more widespread distributions that do not extend across the line. Only 43 mammal species and 97 bird species had a η*_kg_* value between .01 and .9 for at least one of the motifs, *c*.* *4% of the total diversity of each group. These are either (a) species that span Wallace’s Line or (b) species that are confined to locations with mixed Asian and Australian membership. Given the paucity of the latter (mixed red and blue pies, Figure 1), this list mostly represents species with populations on both sides of Wallace’s Line. For mammals, nearly all of these species are bats (Chiroptera), with the addition of two civets (Carnivora), one primate (*Macaca fascicularis*), and four human commensals (three rodents, Rodentia, and one shrew, Eulipotyphla). The 97 species of birds with mixed memberships between the two motifs represent 18 different orders of birds, including highly mobile groups such as waterfowl, birds of prey, shorebirds and waterbirds. These avian orders are: Galliformes (4 species), Anseriformes (3), Podicipediformes (1), Columbiformes (5), Caprimulgiformes (4), Cuculiformes (3), Gruiformes (6), Procellariiformes (4), Ciconiiformes (2), Pelecaniformes (7), Suliformes (4), Charadriiformes (10), Strigiformes (2), Accipitriformes (6), Coraciiformes (4), Falconiformes (3), Psittaciformes (1), Passeriformes (28).

Distinctiveness of the two motifs is born out in an analysis of beta diversity and phylogenetic beta diversity, where we consider those species belonging to one motif with η*_kg_* = 1, and the other with *η_kg_* = 0. Both birds (UniFrac = .838, Bray–Curtis = .895) and mammals (UniFrac = .834, Bray–Curtis = .900) show strong differences in species’ contributions. For reference, a UniFrac (phylogenetic) or a Bray–Curtis (non‐phylogenetic) value of 1 represents entirely non‐overlapping groups, in the underlying phylogeny (i.e. monophyletic clades) or in species composition, respectively. Values for Asian birds are Faith’s PD = 12,090 Myr, *n* = 783; standardized PD = −10.48 *SD*; Australian birds PD = 17,346 Myr, *n* = 1,196, standardized PD = −6.84 *SD*; Asian mammals PD = 5,020 Myr, *n* = 553, standardized PD = −5.59 *SD*; Australian mammals PD = 5,765 Myr, *n* = 415, standardized PD = −7.86 *SD*. All these values are significantly less than expected from random draws over the entire phylogeny, confirming differences between the two motifs. In both mammals and birds, PD is higher in the Australian motif. The oldest lineages with deep evolutionary origins are entirely contained in the Australian fauna – cassowaries and emus in the bird order Casuariiformes and monotremes (Monotremata) in mammals. In the subset of species analysed here, the diversity of these groups is entirely restricted to Australia and New Guinea, anchoring the phylogenetic diversity of these locations deep in the bird and mammal trees.

### 3.2 Further partitions

**FIGURE 2:**
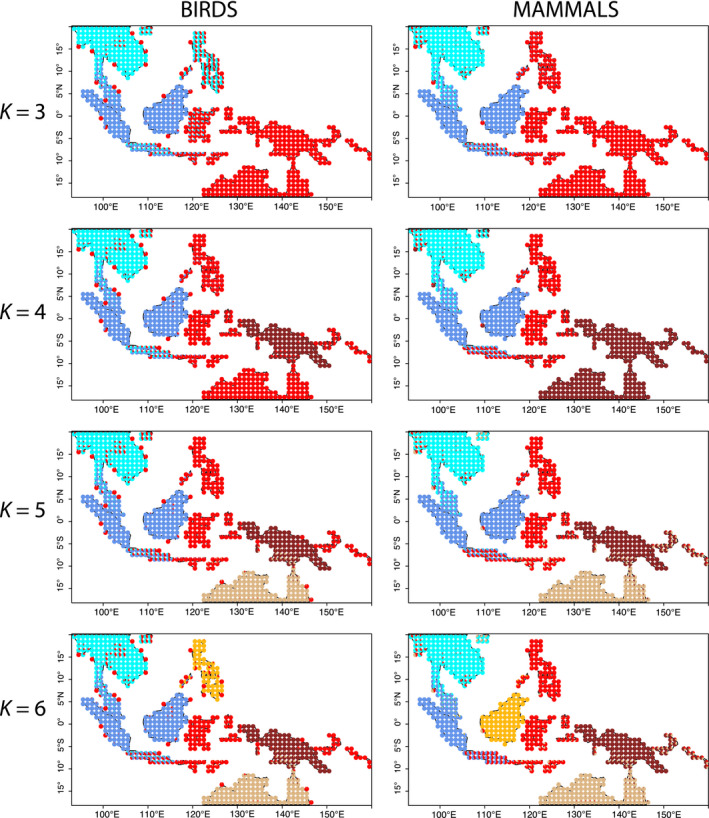
Species motifs for birds and mammals in the Indo‐Pacific, *K* = 3…6. Each pie chart is coloured according to contributions to the location from the inferred biotas. Birds and mammals were analysed separately (colours represent unique groups in each map). Maps for *K* = 7…10 can be found in Supporting Information Figures S2, S3

A persistent question is the extent to which intermixing in Wallacea (the islands lying between Wallace’s original line and Lydekker’s Line, Figure 1) implies that Wallacea should be considered as a distinct zoogeographical region. We asked how partitions naturally arise by considering *K* from 3 to 10 (Figures 1, 2, Supporting Information Figure S2). The first additional subsets are not informative with respect to Wallacea, because they separate continental Southeast Asia from the Sunda Shelf, and, for birds with *K* = 4, New Guinea from everywhere else (Figure 2). However, with *K* = 4 for mammals and *K* = 5 for birds, Wallacea appears as its own motif (Figure 2). For birds, that motif includes not only Wallacea, but also New Britain and the Solomon Islands, lying to the east of New Guinea, and a small representation in central peninsular Thailand. This disjunct motif persists all the way up to *K* = 10 (powder blue in Supporting Information Figure S3). The top 10 species contributing to this motif (when *K* = 10) belong to highly dispersive orders, many of which are strongly linked to coastal habitats: Columbiformes, Procellariiformes, Pelecaniformes, Suliformes, Charadriiformes and Accipitriformes. They include shearwaters, herons, boobies, a frigatebird, a tern, a pigeon and a hawk – all either waterbirds, seabirds, or species with high dispersal ability associated with foraging across large areas and all of which breed in colonies, excepting the hawk. Mammals show no such pattern, and nearly all of the mammal assemblages reflect the contribution of geographically contiguous motifs.

From *K* = 3 to *K* = 6 (Figure 2), Java and Bali appear as a transition zone across Wallace’s Line for both birds and mammals. Nevertheless, a sharp turnover between Bali and Lombok remains, because Lombok is largely populated by Wallacean and Australian elements.

### 3.3 Lineages

**FIGURE 3:**
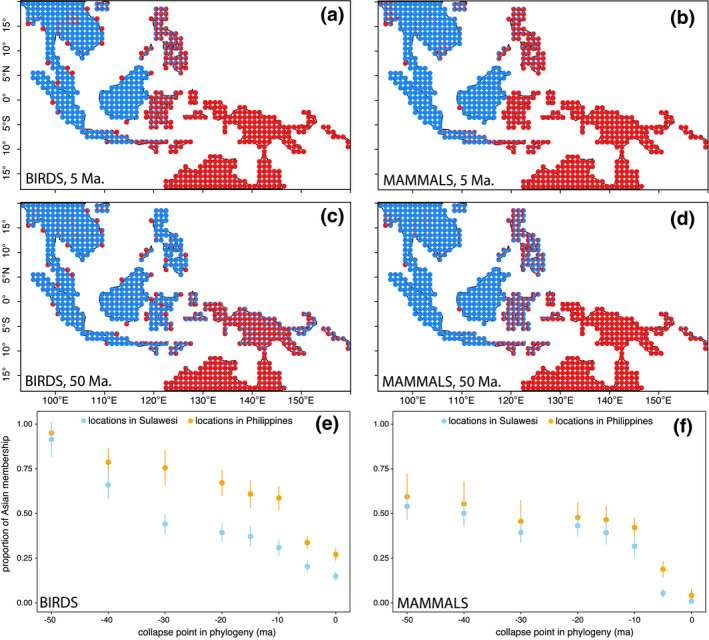
Phylogenetic motifs for birds (left column) and mammals (right), *K* = 2. Each pie is coloured according to the contributions of the regional biotas after accounting for relationships at different time points in the phylogeny. (a,b) Motifs generated by subtending the tips of the phylogeny at 5 Ma. (c,d) Motifs generated by subtending the tips of the phylogeny at 50 Ma. (e,f) Mean ± *SD* for the average composition of the map cells in the Philippines and Sulawesi at different time points (additional maps showing time points are in Supporting Information Figure S4)

Incorporating phylogeny, we find Asian and Australian biotas (*K* = 2) show increased levels of mixing and interdigitation across Wallace’s Line as one considers clades at higher taxonomic levels. Surprisingly, we also find that the essential division between the two motifs moves east with deeper lineages (Figure 3, Supporting Information Figure S4). At the present day, for birds, Sulawesi and the Philippines largely fall into the Australian motif, with the Australian biotas contributing about 85% to the Sulawesi avifauna and 75% to the Philippine avifauna (Figures 1, 3e). For mammals, Australian affinities are even stronger (Figures 1 and 3f). At 50 Ma, where just the 30 mammal lineages and 59 bird lineages are retained in total, the situation is reversed: for birds the Asian biota now contributes almost entirely to both the Sulawesi and the Philippines, and for mammals it contributes 60% (Figure 3, Supporting Information Figure S4). In Figure 3e and f we show for Sulawesi and the Philippines how the contributions from the two biotas are altered as one moves deeper in the tree. For birds, especially in Sulawesi, the Asian lineages come to predominate deep in time. For mammals, approximately equal contributions of each motif have accumulated by 10 Ma, suggesting that the inferences of an Australian make‐up at the present‐day result from a greater number of wide‐ranging species extending to Sulawesi and the Philippines from the east rather than the west, but similar numbers of wide‐ranging genera and families extend from both the east and west. Maps for each time slice between 10 and 50 Ma are shown for *K* = 2 in Supporting Information Figure S4.

## 4 DISCUSSION

In this paper, using a method that assigns species to biotas based on their co‐distributions and proportional contributions of biotas to map cells, we have documented the extent of biotic mixing across Wallace’s Line. A feature of the method is that it does not require biogeographical units to be contiguous (Valle et al., 2018), and we identify such examples. Nevertheless, we find strong support for a line that corresponds to Huxley’s modification of Wallace’s original line, except that Palawan is closely associated to the Philippines in its biogeographical history (as inferred also by Esselstyn et al., 2010). The boundary is generally sharp, especially between Borneo and Sulawesi, but with admixture of biotas in the Philippines for birds, and in Java for mammals. Sulawesi is placed east of the line, but when we consider deep lineages rather than species (approximately at the ordinal level for birds), Sulawesi moves to the west of the line for birds, and becomes admixed for mammals. Finally, above certain values of *K*, Wallacea is reasonably considered as a distinct zoogeographical region, but is linked to disjunct coastal habitats and small islands, providing one example of a disjunct biogeographical region. Weber’s and Lydekker’s lines (Figure 1) lie within Wallacea. When we set *K* to higher values (i.e. divide the region into smaller subsets), we confirm these lines correspond to zones of transition, with components from a number of zoogeographical regions (Supporting Information Figures S2, S3). All these results are unified by considering that the islands of Wallacea have been always separated by water gaps from the mainland (Voris, 2000; Supporting Information Figure S5), and have a relatively seasonal, drier climate (Supporting Information Figure S5), both of which generate a suite of dispersive species. We suggest these species fail to penetrate the recently connected parts of the Sunda shelf because of the established less dispersive fauna there.

Ficetola et al. (2017) invoke climate, topography, and tectonic plate movements as underlying causes of the boundaries delineating major realms within the northern continents, and climate as important in the setting of boundaries of zoogeographical regions nested within these realms. Climate and topography are thought to delineate realm boundaries by limiting dispersal of whole faunas. Plate tectonics is invoked as bringing together historically disjunct and independently evolving faunas, and identified as especially important across southern Eurasia. The three factors considered by Ficetola et al. (2017) have also been considered as explanations for Wallace’s Line. First, climate differences across the line are small, but they do continuously vary from a drier and more seasonal climate in Wallacea, to year‐round wetter conditions further west (Supporting Information Figure S5), which van Welzen et al. (2011) suggested is the reason for a westward extension of Wallacean plants across the line into Java. Second, plate tectonics has been invoked because parts of Sulawesi were formerly connected to Asia and other parts to Australia. The Sula spur detached from Australia and moved northwards, colliding with the Asian plate at *c*.* *20 Ma (Hall, 2017), and small fragments were likely continually above water during this time (Nugraha & Hall, 2018). Transport of Australian taxa by plate movements has been most successfully invoked to explain presence of a freshwater snail (von Rintelen et al., 2014), but bird and mammal colonization of Sulawesi post‐date island movements (Stelbrink et al., 2012). For example, the two marsupials on Sulawesi (dwarf cuscus, *Strigocuscus celebensis*, and bear cuscus, *Ailurops ursinus*), which are themselves sisters, diverged from their nearest Australian relatives *c*. 12 Ma (Mitchell et al., 2014). Given climate and tectonic movements seem to account for a relatively small fraction of the biogeographical pattern, we consider dispersal to be the primary mechanism of range expansions across the region and dispersal limitation as the cause of Wallace’s Line (Lohman et al., 2011; Stelbrink et al., 2012).

### 4.1 Position and width of the line

Sulawesi has always formed a major point of contention about the position of Wallace’s Line. Wallace himself was uncertain about whether the island should lie to the east of the line (as he originally proposed) or to the west of the line (in his later writings, van Welzen et al., 2011). Modern‐day faunal studies have placed the boundary to the east of Wallace’s original line. These include assessments based on qualitative observations of species (van Oosterzee, 1997), on distance methods (Escalante, 2017; Holt et al., 2013) and network approaches (Edler et al., 2017). Consequently, Lohman et al. (2011, p. 209) concluded ‘there is now little doubt that the bulk of Sulawesi's fauna is of Asian origin’. For example, out of the 133 mammals presently on the island (https://www.worldwildlife.org/ecoregions/aa0123), only the two marsupials have clear Australian connections. Even deeper in the phylogeny, at the level of higher taxa, the disparity is weakened because of many endemic, albeit predominantly Asian, radiations (Driller et al., 2015; Hawkins et al., 2016; Stelbrink et al., 2012). At 50 Ma, 11 mammal lineages are present in Sulawesi, of which one is Australian.

Contrary to these observations, when considering the present distributions of species, we find Sulawesi is linked to the Australian biota, not Asia (Figure 1), in agreement with the original inferences of Wallace. The difference between our results and the other recent quantitative assessments arises because the method we employ emphasizes co‐distributed species over an entire realm, so island endemics, including those on Sulawesi, make little difference to delineating realm boundaries when we set the number of biotas to be low (e.g. *K* = 2 or 3). Other distance methods explicitly include endemics in calculating distances between map cells (note the network analysis so far applied to mammals relied on observational records and is likely affected by the distribution of observations, Edler et al., 2017). The linking of Sulawesi to Australia in the present paper arises because species on Sulawesi have ranges extending east. However, when we consider deeper taxonomic levels, Sulawesi becomes associated with Asia. Lineages have greater spatial extent than species, and consequently a number of Asian lineages extend into Sulawesi, thereby drawing it into the Asian motif. For example, a single species of woodpecker is found on Sulawesi, which belongs to a lineage containing 60 species in Asia, but none east of Sulawesi.

### 4.2 Wallacea’s origins through dispersal

The main features of Wallacea are the continued presence of water gaps throughout time, whereas water barriers disappeared across the Sunda shelf and across the Sahul shelf during the glaciations (Ali et al., 2020; Voris, 2000). Importantly, water gaps on the Sunda shelf may have been absent for long periods before about 400,000 years ago (Husson et al., 2019. These features, together with a relatively dry and seasonal environment (Supporting Information Figure S5; van Welzen et al., 2011) appear to have driven the formation of a uniquely dispersive Wallacean biota, with ecological traits indicative of high dispersal ability and potential for successful population establishment across Wallacea. Van Welzen et al. (2011) identified several plant families and a life‐form (herbaceous, high dispersal abilities) reflecting a Wallacean ecological syndrome. They suggested this syndrome reflects the regular crossing of water gaps over the past few million years, as well as the more seasonal environment. Distant island assemblages similarly reflect the influence of dispersal. The fauna of Christmas Island, a small oceanic island *c*. 350 km south of Java, is linked to Wallacea (Ali et al., 2020), and the Nicobar Islands contain at least one species with Wallacean affinities (the Nicobar megapode *Megapodius nicobariensis*). These islands have never been connected to the mainland.

The term dispersal has been used in two ways, as movement to a new place, or as both movement and colonization. When contrasted with vicariance, the term implies movement followed by colonization, that is, a range expansion (Borregard et al., 2016; White, 2016). Reviews of the literature, and global analyses of a large bird data set, confirm that both rate of arrival in a new location, and ease of establishment once arrived, are demonstrably important to range expansions (Price, 2008, ch. 8; Pigot et al., 2018). Rate of arrival depends on a species’ mobility, which may be linked to its ecology, plus the nature of barriers (White, 2016). Establishment once arrived is promoted by propagule size, and affected by abiotic and biotic conditions in the recipient location (Borregard et al., 2016; Gillespie & Roderick, 2002; Mayr & Diamond, 2001, ch. 11). Features more specifically thought to increase probability of dispersal between islands include occupation of coastal habitats (Mayr & Diamond, 2001, ch. 7; Ricklefs & Bermingham, 2002), abundance (Mayr & Diamond, 2001, ch. 7), feeding generalism, and flocking habit (reviewed in Price, 2008, ch. 8). Some species regularly move between islands for feeding or migration. Several of these features can be seen in the motifs. For example, one motif spans coastal and wetland habitats across the entire region (including a small contribution of this motif to locations in central Thailand, see top left panels in Supporting Information Figures S2, S3), bats are the mammal species that most frequently cross Wallace’s Line, and mammals of east Java may be linked to Wallacea by its relatively drier and more seasonal climate (and associated flora, van Welzen et al., 2011). These features are reflected even at the lowest level of partitioning (Figure 1, red), and appear more strongly reflected by birds, further implicating dispersal capability as a mechanism driving biotic assembly in the region. Though the presence of a shared motif between the mainland in Southeast Asia and Australia (Figure 1, red) appears first to merely reflect biotic connections with the mainland and the Philippines and Sulawesi (Figure 2, red), higher values of *K* (Supporting Information Figure S2, top panels of Figure S3) also link smaller distant islands with mainland Southeast Asia (as well as the Philippines and Sulawesi), likely driven by the dispersal syndromes indicated above.

Both rates of arrival (i.e. movements) and possibilities for establishment once arrived are likely to apply especially to a few species, making them good candidates to broadly range across Wallacea. Kennedy et al. (2017) identify wing pointedness, a correlate of mobility, as importantly contributing to the colonization of small islands by corvoid birds. A network analysis identified four biogeographical ‘modules’ within Wallacea, which are united by isolation, area and elevation (Carstensen et al., 2012), features expected to affect turnover and repeated range expansions for more mobile species. Turnover may be amplified because some islands were historically small, and regularly disturbed. Notably, geological evidence indicates that at 10 Ma Sulawesi may have consisted of just three islands, whose total area was less than 7% of that of current Sulawesi. It also experienced significant volcanic activity and mountain building in the past two million years (Nugrha & Hall, 2018). The multiple causes of wide ranges, coupled with a large stochastic element, mean that a list of species with large ranges across an archipelago is often idiosyncratic, with no single uniting attribute (Mayr & Diamond, 2001, ch. 7). For birds, using the *ExtractTopFeatures* function in *ecostructure*, we found 24 bird species that with *K* = 2 are confined to the Australian motif, are widespread across Wallacea, and are present in Sulawesi, thereby importantly linking Sulawesi to the Australian biota (Supporting Information Table S1). They exemplify many of the diverse mechanisms driving range expansions across water gaps, including mobile species such as two swifts (*Aerodramus* spp.), migratory species such as a cuckoo (*Scythrops novaehollandiae*), abundant species in lowlands such as the island monarch (*Monarcha cinerascens*), and an egret (*Egretta picata*), which is likely to regularly visit very small islands (see Mayr & Diamond, 2001, ch. 7).

The 24 bird species just discussed do not extend west beyond Wallace’s Line, which appears contrary to their inferred high dispersal abilities. We argue the best explanation for this is competition and predation by incumbents, which are well established as a limiting factor in the colonization of islands (the best evidence comes from rapid colonization of defaunated islands, coupled with long persistence times of established species sometimes reflecting the age of the island; Borregard et al., 2016; Gillespie & Roderick, 2002; Waters, 2011). Species established on the Sunda Shelf islands may be at a competitive advantage to those dispersed through Wallacea, not only as a result of incumbency effects, whereby small arriving propagules experience large resident populations, but also because they are long established in a more continental community. This may apply to plants too, given the strongest westerly barrier is the original Wallace–Huxley Line, which sets the range limits of > 3,000 species (van Welzen et al., 2011). Predation is likely also to be important in limiting establishment, with several predatory species, such as the Bali tiger, *Panthera tigris*, only present west of Wallace’s Line, whereas the most fearsome predator on Sulawesi is a civet cat. Predation by continental species introduced to remote islands has been a major cause of recent island extinctions (Fritts & Rodda, 1998).

### 4.3 Modelling considerations

The results and conclusions we draw here rely on an imperfect knowledge of species presences, uncertainty in phylogenetic tree construction, and probabilistic methods that feed such uncertainty forward to generate the geographical patterns we interrogate. Nonetheless, the admixture‐based method and visualization we employ highlight nuanced geographical differences in biotas in a single interpretable Structure plot visualization; such interpretability is difficult to achieve with other dimension reduction methods such as principal components analysis (PCA) or multidimensional scaling (MDS) (see White et al., 2019 for discussion). One limitation of our approach is that it may yield different models across different values of *K* and even across different model runs for the same value of *K*. Here, for each value of *K*, we report the results for the best fitted model across 10,000 runs with different initializations, where model fit is assessed in terms of BIC or Bayes factors (Valle et al., 2014; White et al., 2019).

One might also wish to compare model fit across different values of *K*, but we do not employ such an approach here. While higher values of *K* may highlight finer structure in data, those high values may also lead to biologically unremarkable partitions, such as finding that major islands represent endemic faunas, or produce cluttered and uninterpretable visualizations (Dey et al., 2017; White et al., 2019). Additionally, the population genetics literature argues that a value of *K* in an admixture model estimated by any sound statistical procedure is almost always an underestimate (Lawson et al., 2018). As applied here, the choice of *K* also depends on the species and geographical locations we include in the analysis, not to mention statistical considerations regarding a priori assumptions about the data (see Valle et al., 2014, 2018; White et al., 2019). In a previous global analysis of birds using this method, a partition along Wallace’s Line appears even at the scale of the entire globe at *K* = 11 (White et al., 2019), suggesting the primacy of the bipartition when all bird species and global map cells are included. Our investigation focuses on biotic mixing across Wallace’s Line, prompting a sequential partitioning of the region according increasing values of *K* to interrogate the discrete nature of these partitions. The partitions we analyse here are largely nested, implying results are robust, with connections between disparate locations similarly robust to increased partitioning. We have shown here that many biological insights are gained from a holistic examination of the results for different values of *K*, rather than an assessment based on a single ‘best’ value.

### 4.4 Conclusions

By using a method that separates biotas (collections of co‐distributed species) from the locations populated by the biota, we demonstrate that one of the most striking turnovers in faunas in the world, in the Indo‐Australian Archipelago, is sharp and as generally thought, associated with the most westerly deep‐water channel. We identify how zones of transition can be related to the mobility of species and ecological conditions, indicating the importance of dispersal and dispersal limitation in global bioregionalization. The ability to estimate the proportional contribution of biotas to locations should be useful going forward in understanding in greater depth what sets other realm and zoogeographical boundaries (White et al., 2019). For example, Ficetola et al. (2017) suggest that plate tectonics is important in setting boundaries in Eurasia, which should be detectable through the comparison of relatively old lineages. These analyses can be applied to any geographical scale (Vacher et al., 2020; Valle et al., 2014, 2018). In other applications, the method could be applied to regions of particular conservation value. For example, ecoregions are used to describe discrete biotas at a more local scale, based on both physical and biotic conditions and the extent to which they intergrade (Dinerstein et al., 2017; Olson et al., 2001; Vacher et al., 2020). We propose that these methods are suitable to assess concordance of ecoregions with threatened and endangered species, an additional important issue (Dinerstein et al., 2017; Edler et al., 2017). The ability to simultaneously cluster species and the locations where they are found, whether communities, ecoregions or realms, has great potential to generate insights in ecology and biogeography. Applications extend far beyond those that are explored here.

## AUTHOR CONTRIBUTIONS

A.E.W., K.K.D. and T.D.P. designed the study. A.E.W., K.K.D. and M.S. performed the analyses. A.E.W., K.K.D. and T.D.P. wrote the paper, with input from all authors.

## BIOSKETCH

**Alex White** is a post‐doctoral fellow in the Data Science Lab at the Smithsonian Institute in Washington, D.C., USA. His research focuses on the ecological and evolutionary mechanisms mediating the distributions of species across disparate taxonomic groups including plants and birds.

## Supporting information

Fig S1

Fig S2

Fig S3

Fig S4

Fig S5

Supplementary Material

## Data Availability

Bird biogeographical data are available from Birdlife International and mammal biogeographical data are available from IUCN (http://iucnredlist.org). Code for fitting grade of membership models, analysing motifs, and generating visualizations is publicly available in the R package *ecostructure*. The package contains: (a) functions for combining presence–absence data with a phylogeny to fit phylogenetic motifs, (b) functions for generating presence–absence matrices from GIS data sources to fit the models used in this analysis, and (c) functions for visualizing the output of those model fits. The package, and a detailed vignette of its functionality, is available for download at https://kkdey.github.io/ecostructure
