# Supplementary figures and images for: Dispersal syndromes drive the formation of biogeographical regions, illustrated by the case of Wallace’s Line
Source: Glob Ecol Biogeogr. 2021 Jan 6;30(3):685–96. doi: 10.1111/geb.13250 (PMC7986858; doi:10.1111/geb.13250)

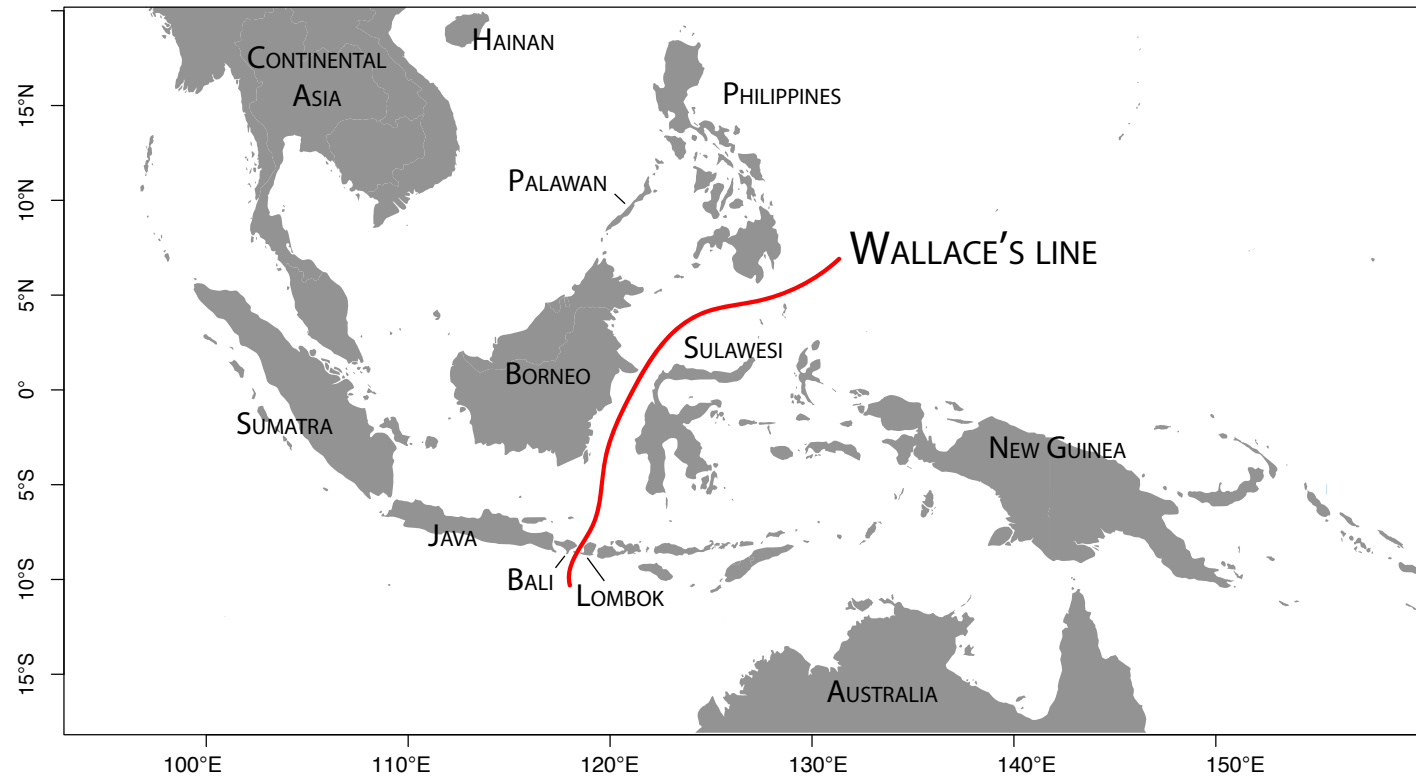

Supplement: Supplementary file 1 — Fig S1 [file GEB-30-685-s005.pdf]

# BIRDS

K=7

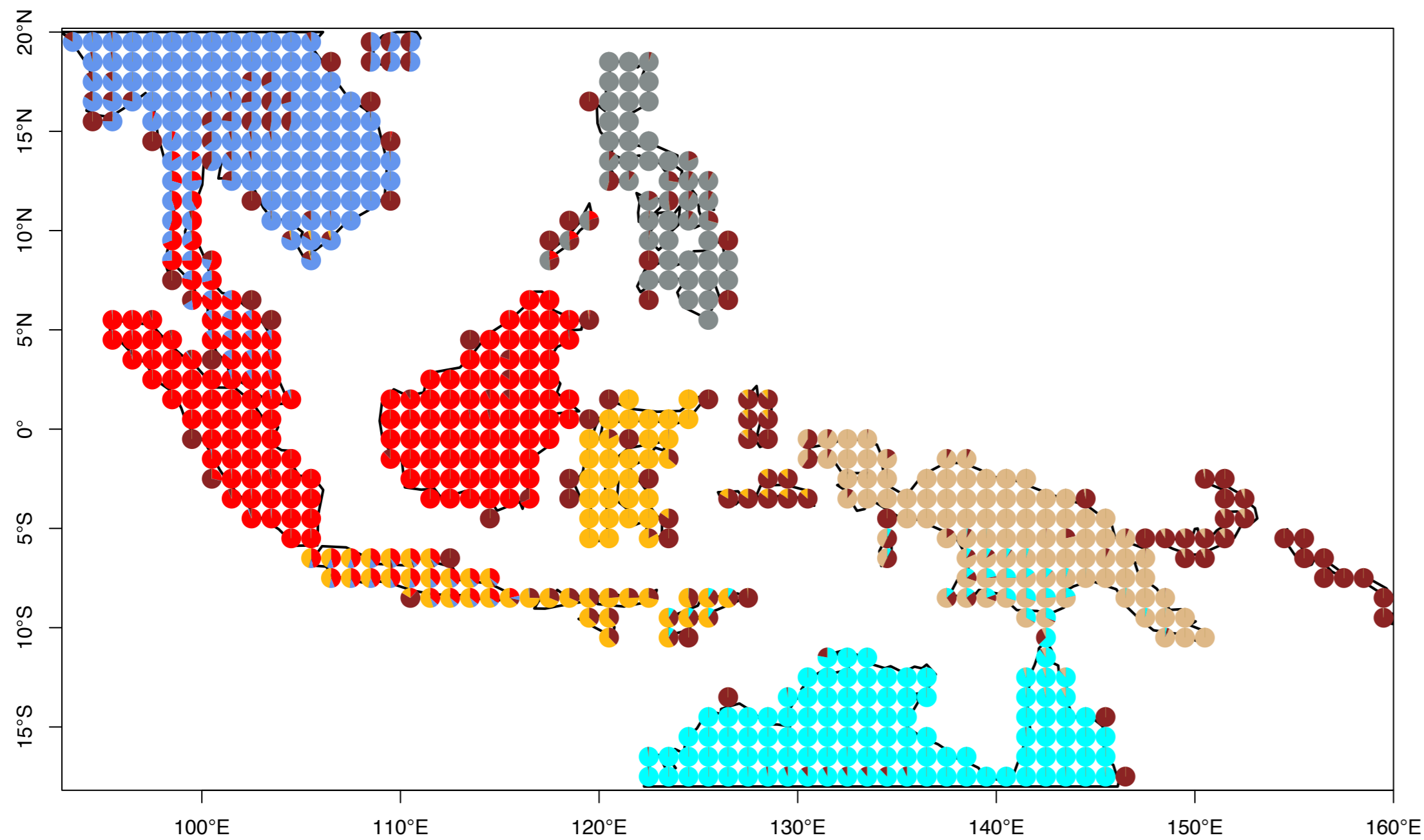

# MAMMALS

K=8

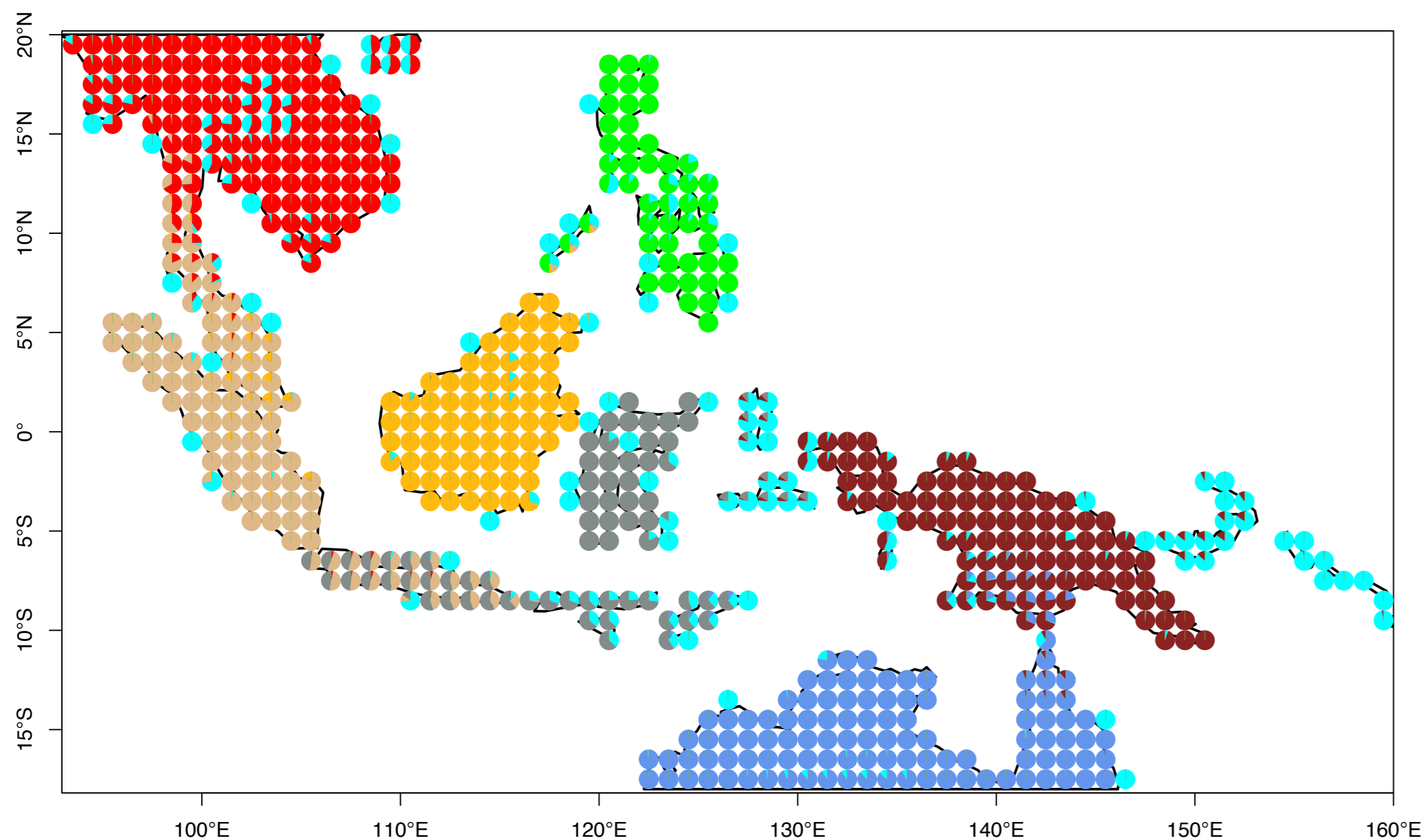

K=9

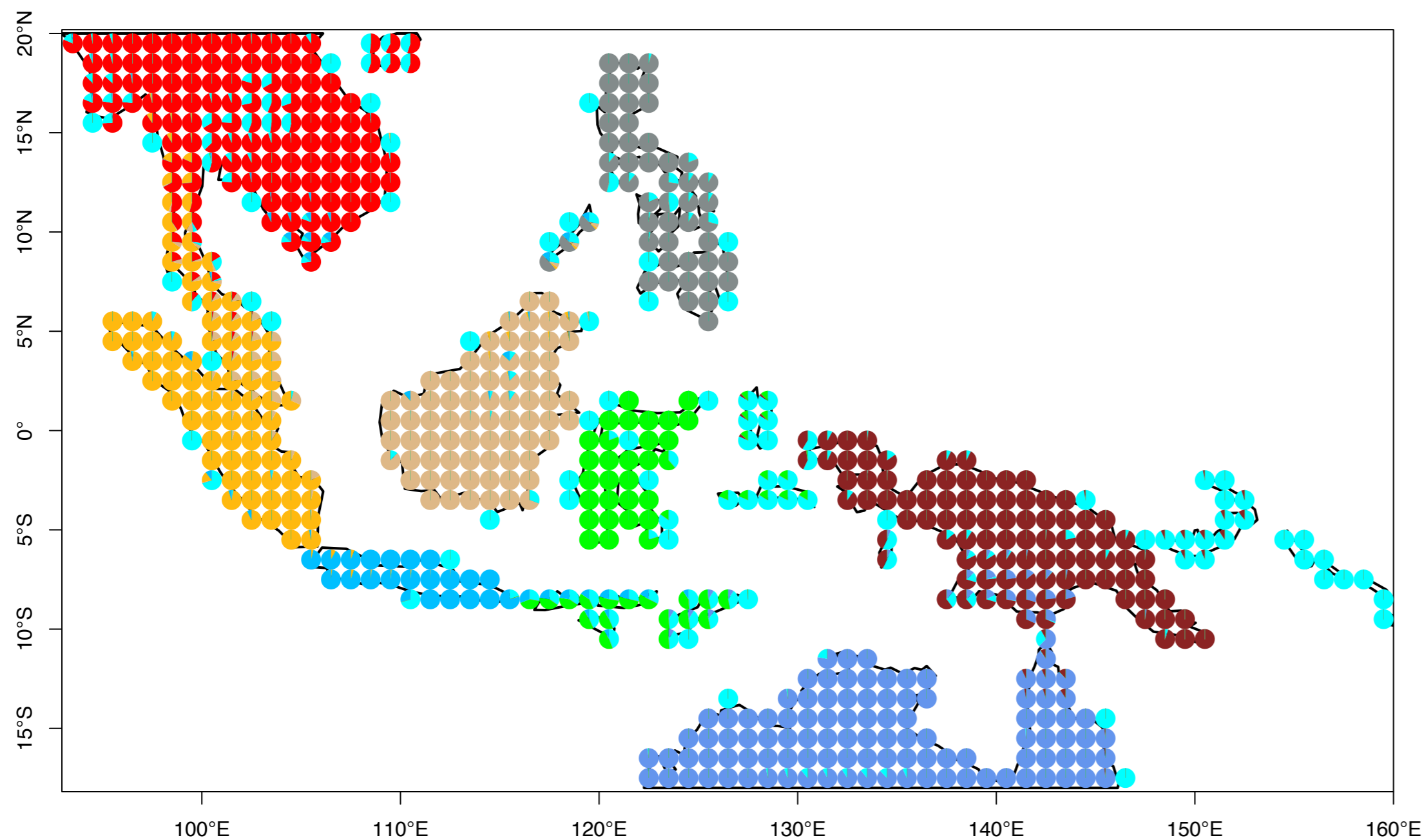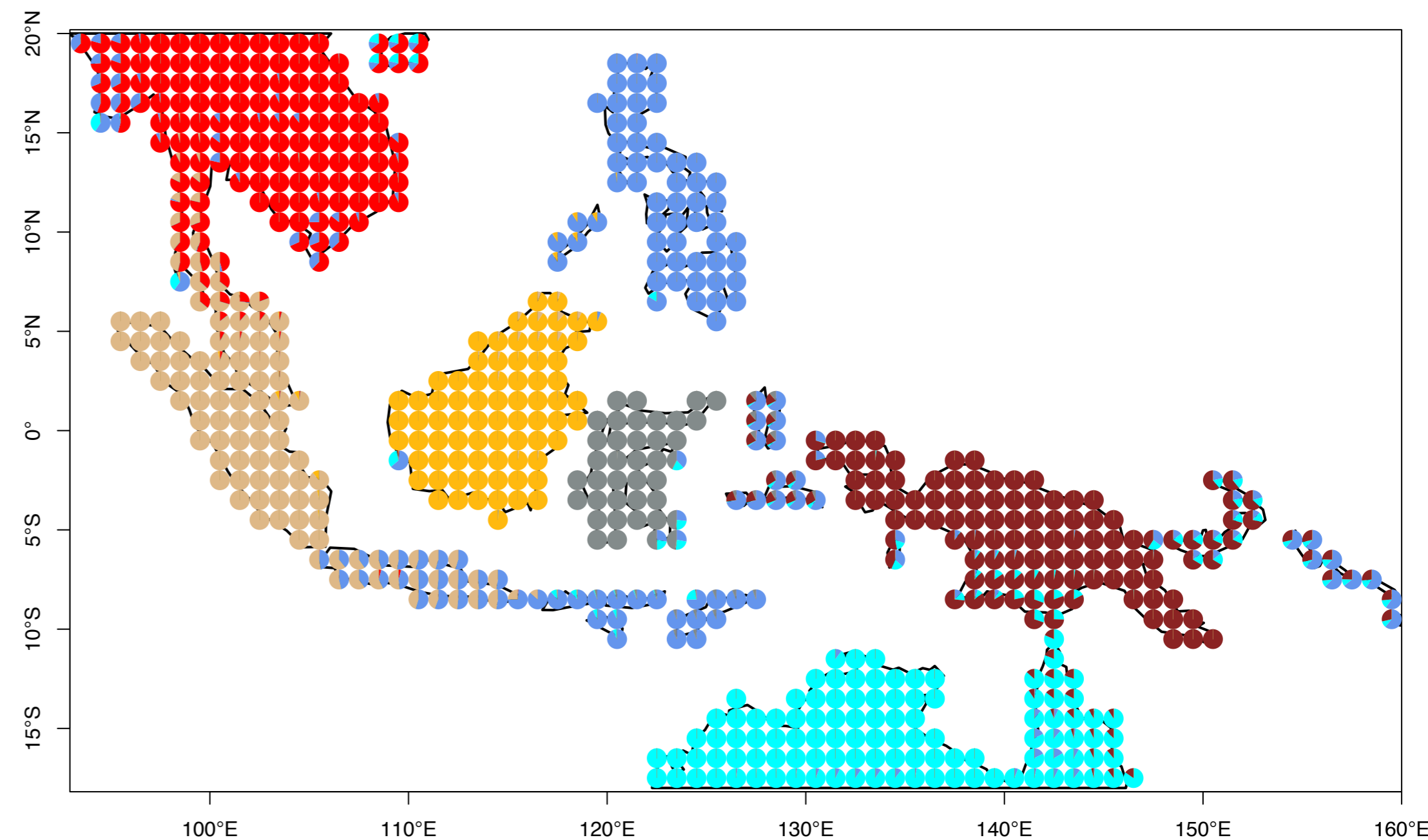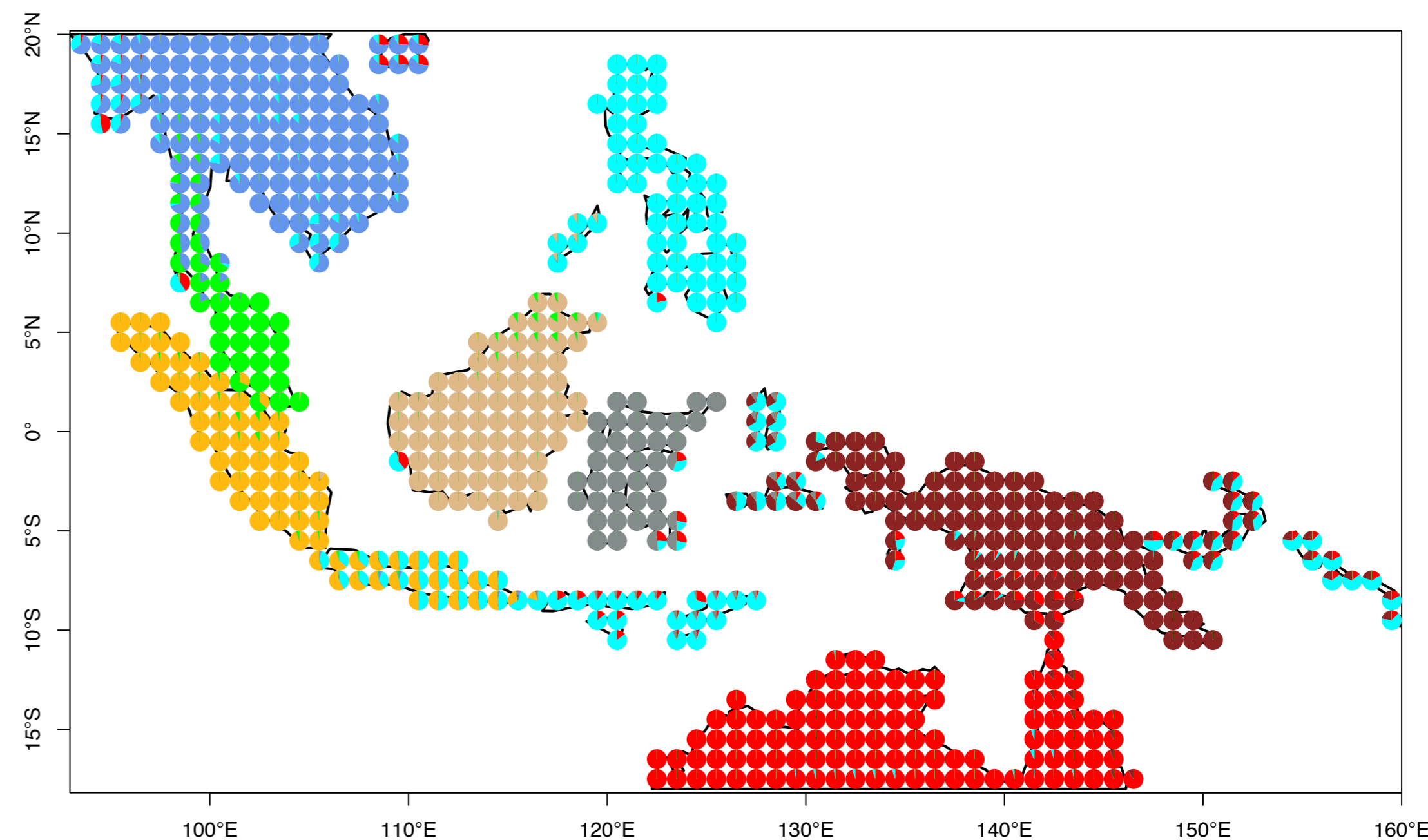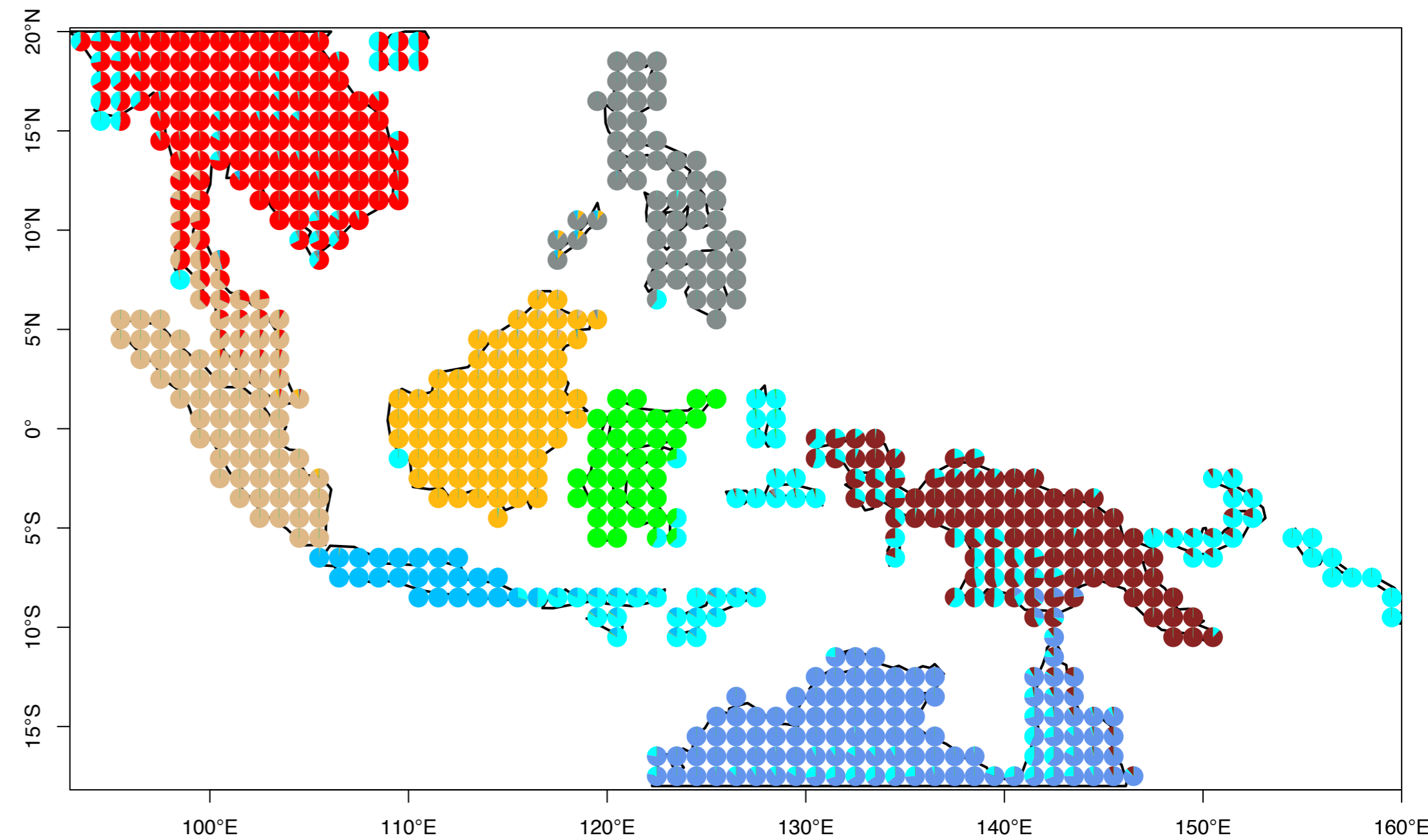

Supplement: Supplementary file 2 — Fig S2 [file GEB-30-685-s004.pdf]

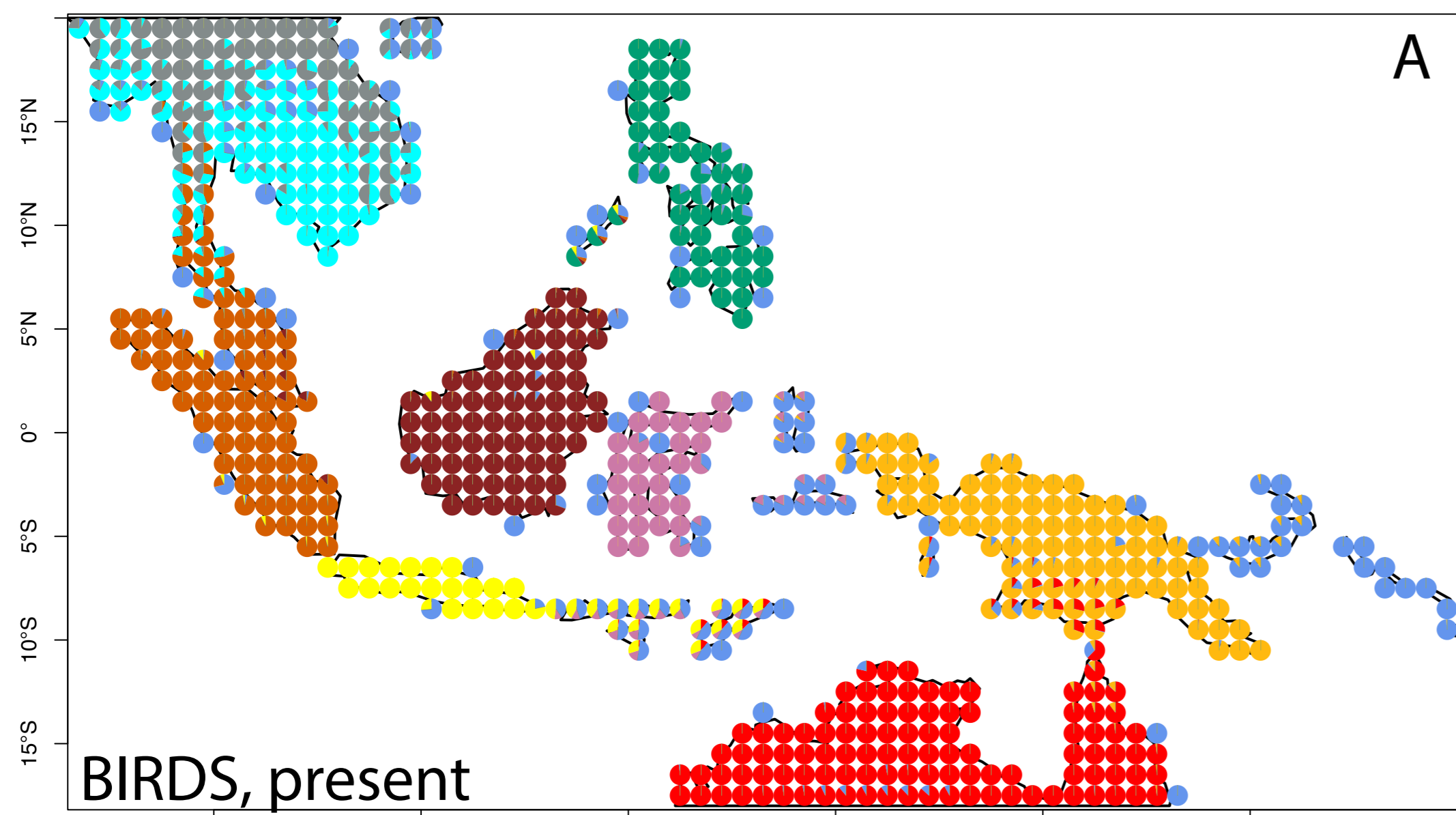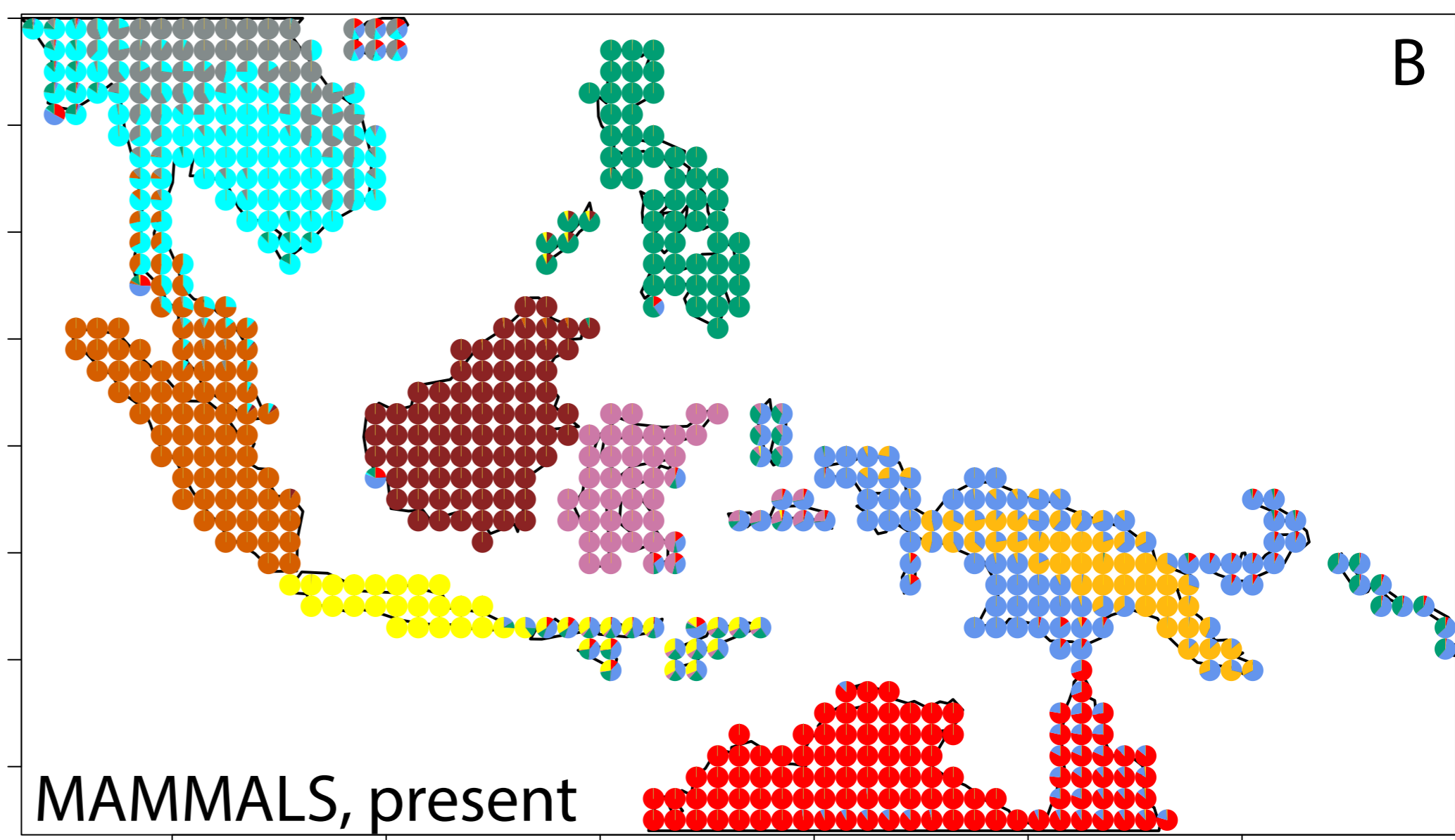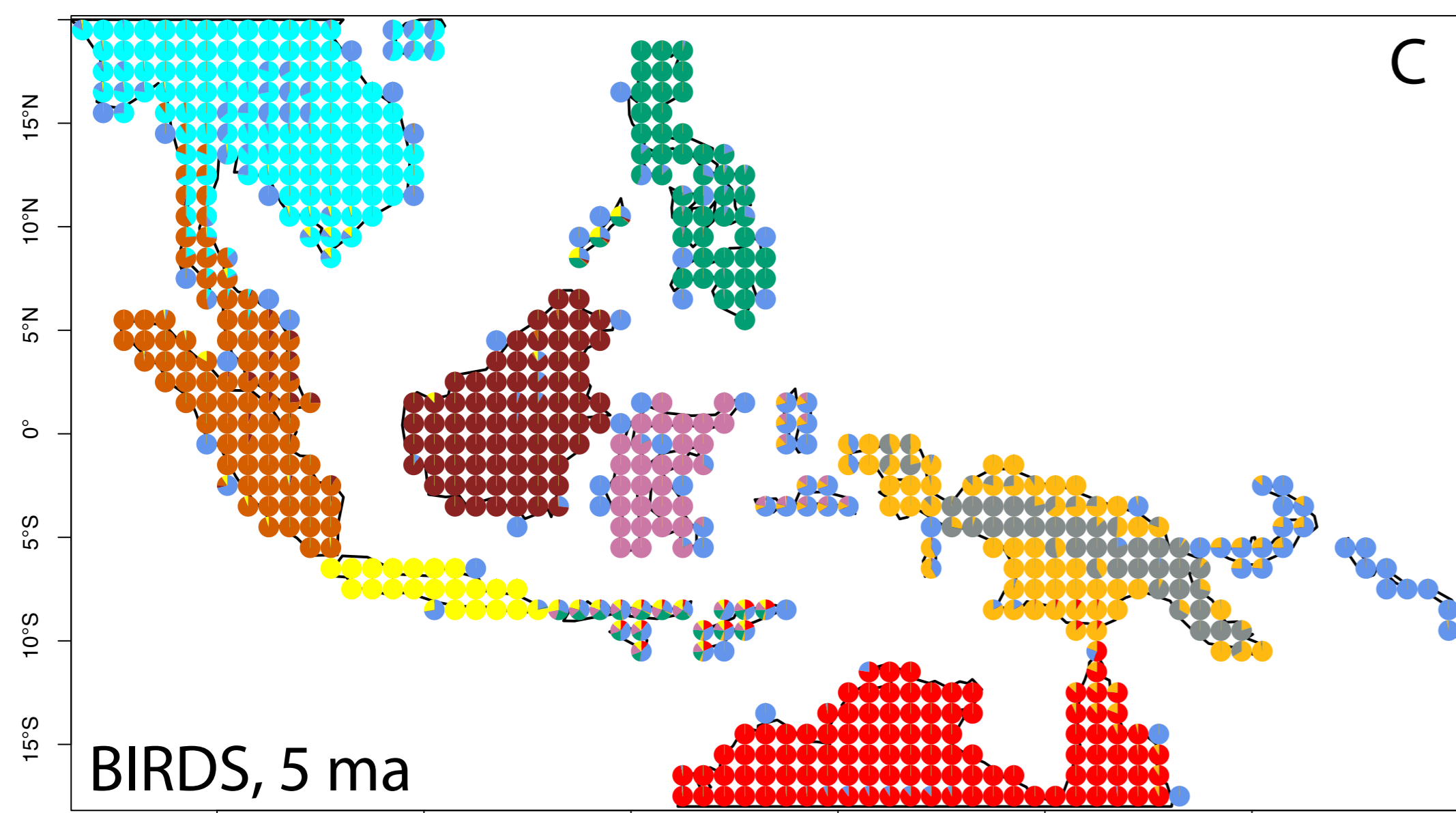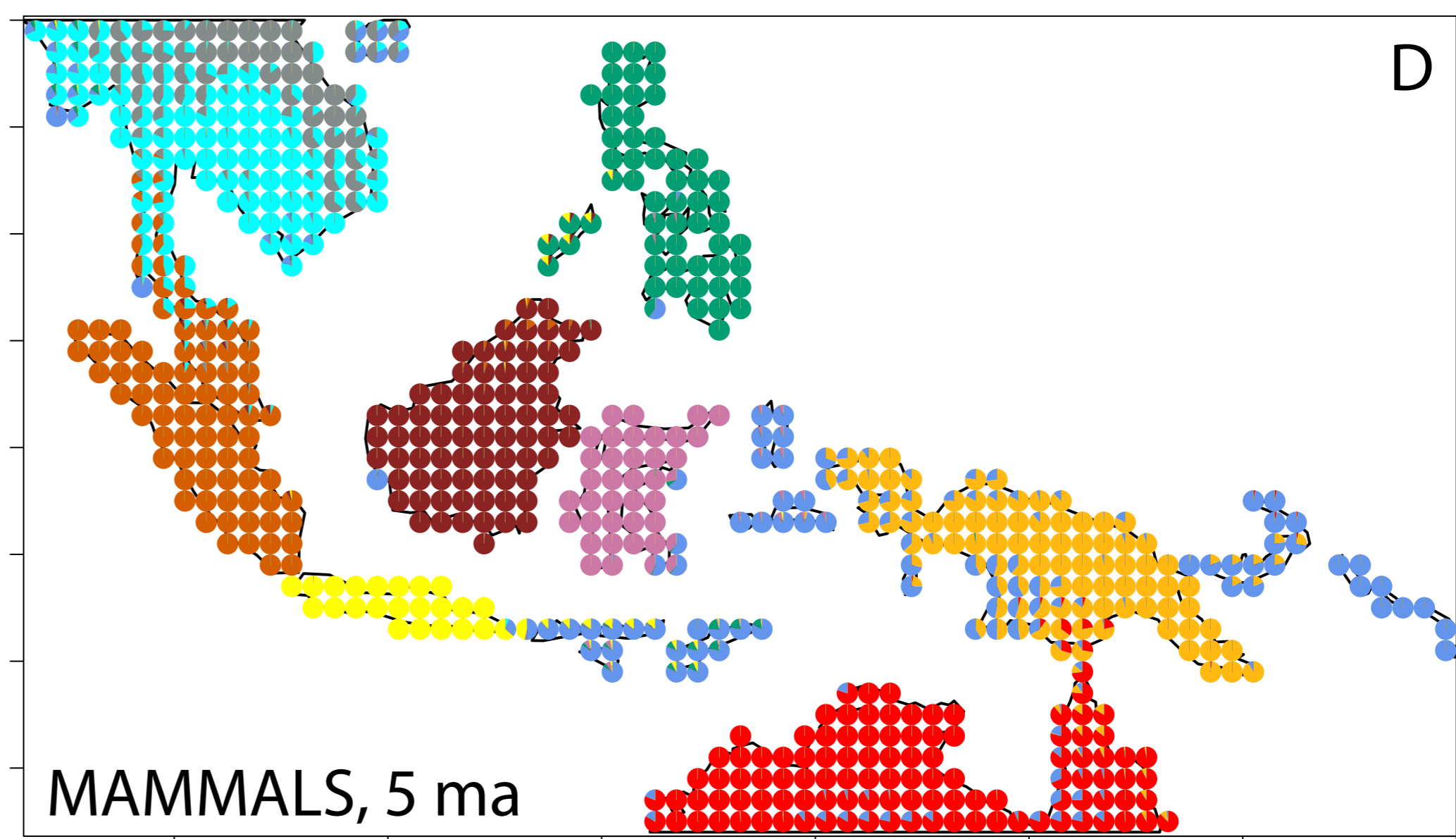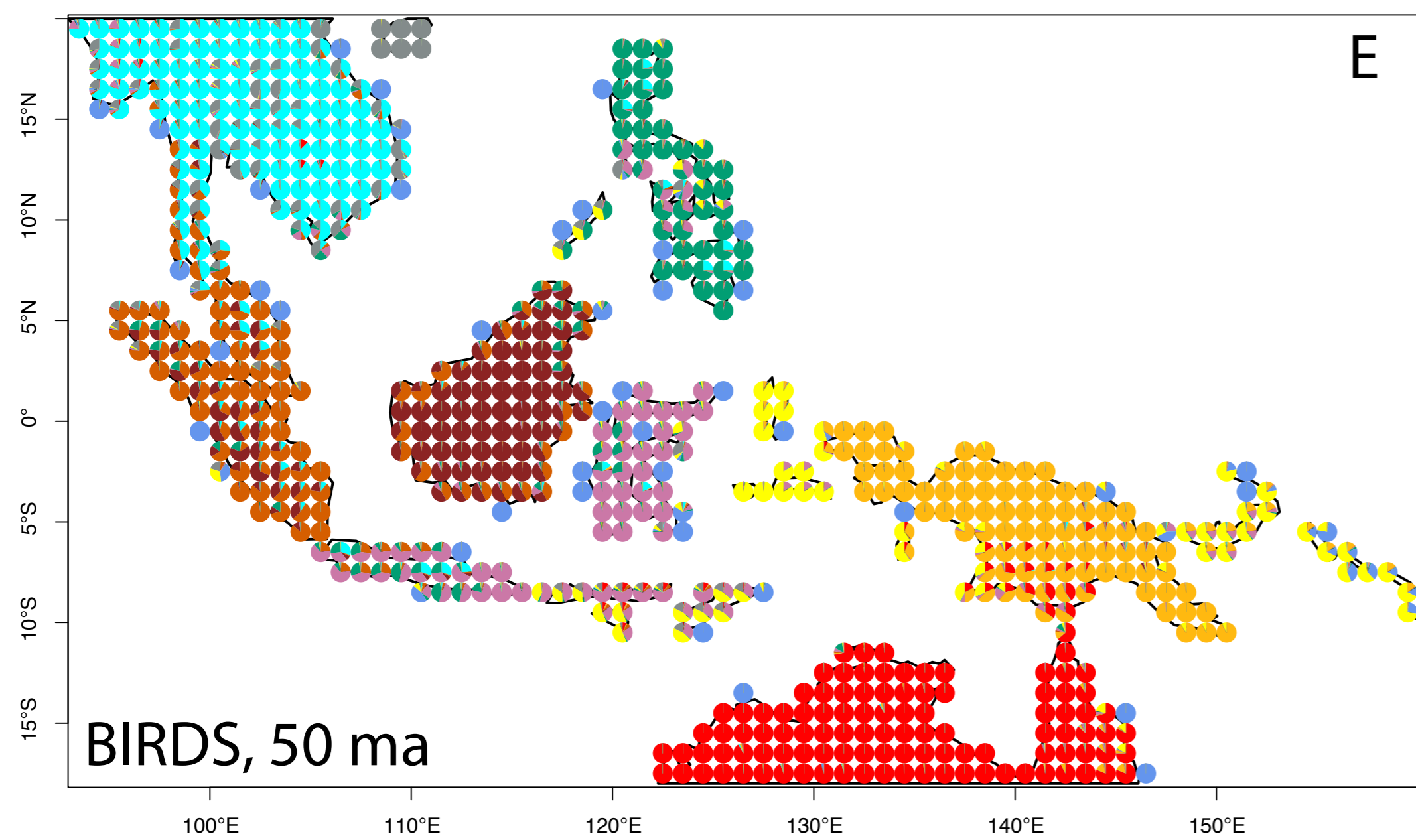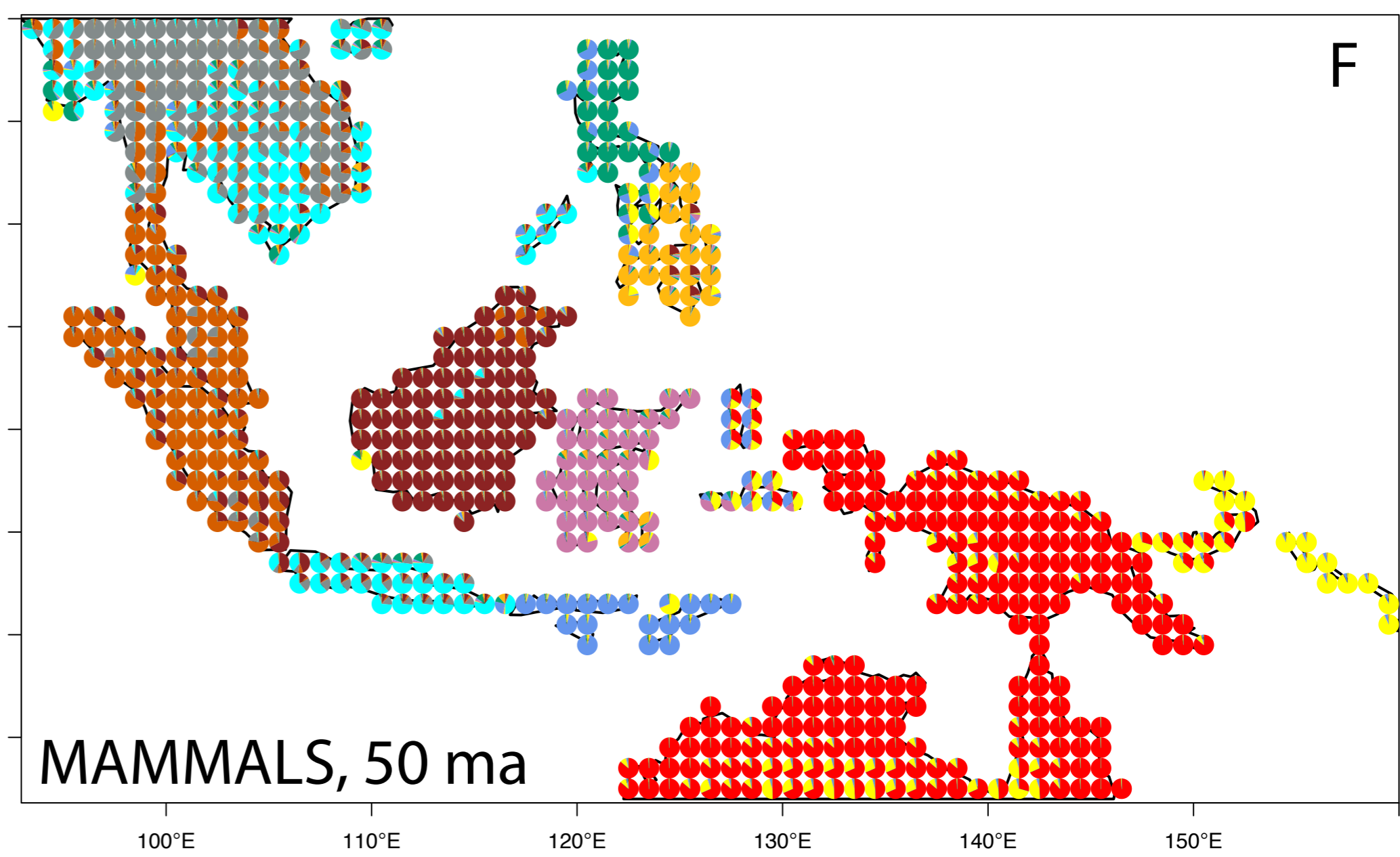

Supplement: Supplementary file 3 — Fig S3 [file GEB-30-685-s003.pdf]

# BIRDS

# MAMMALS

10 ma

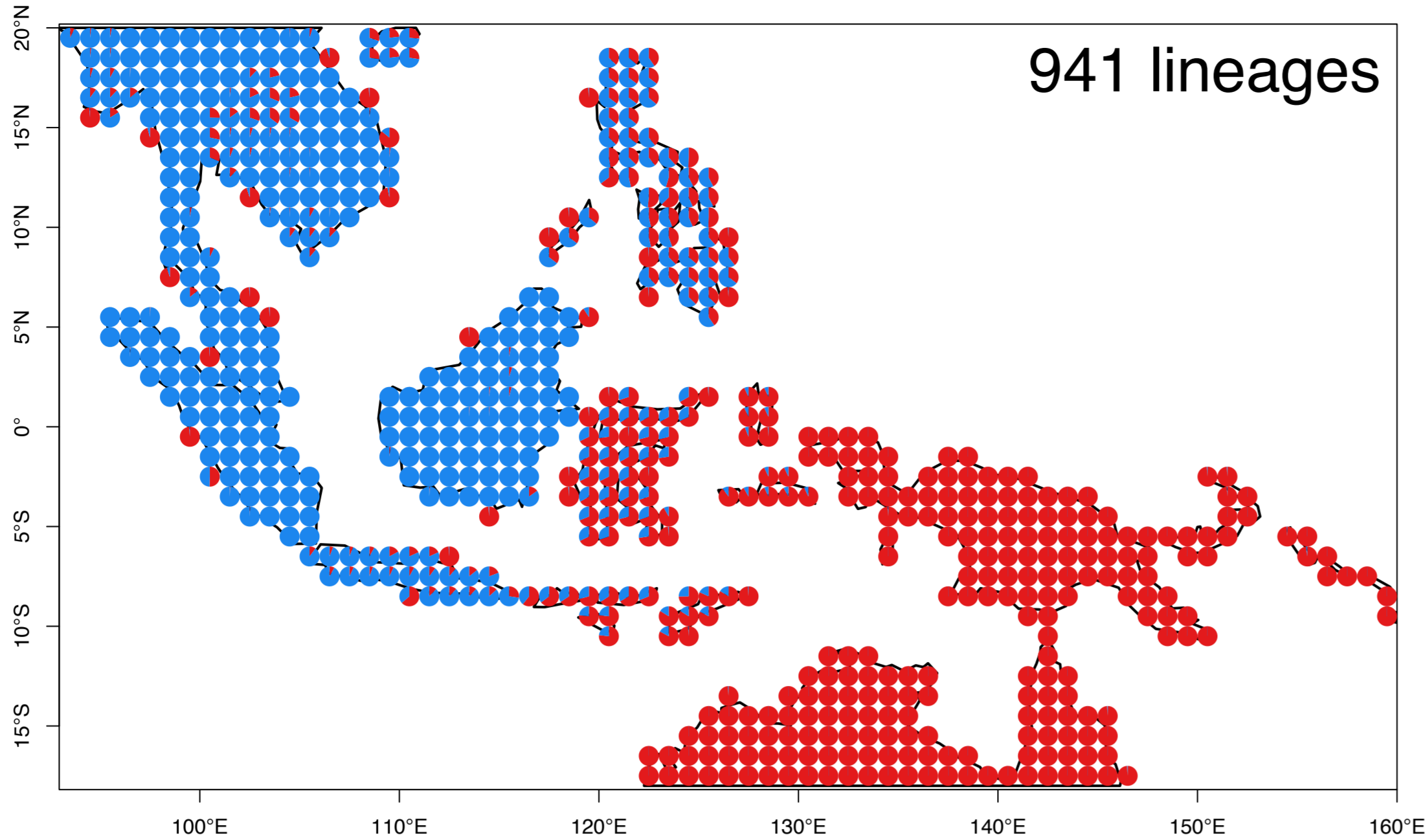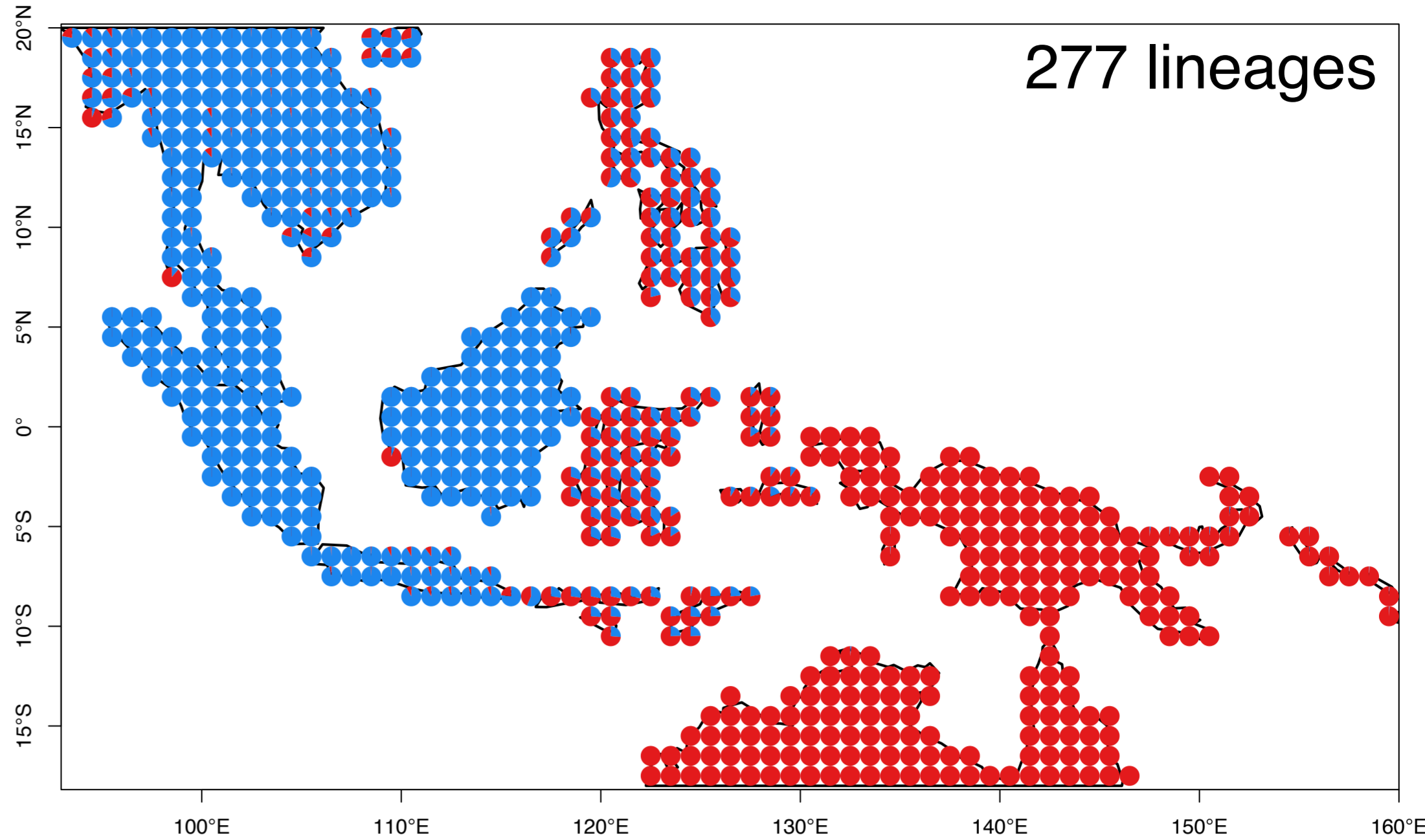

20 ma

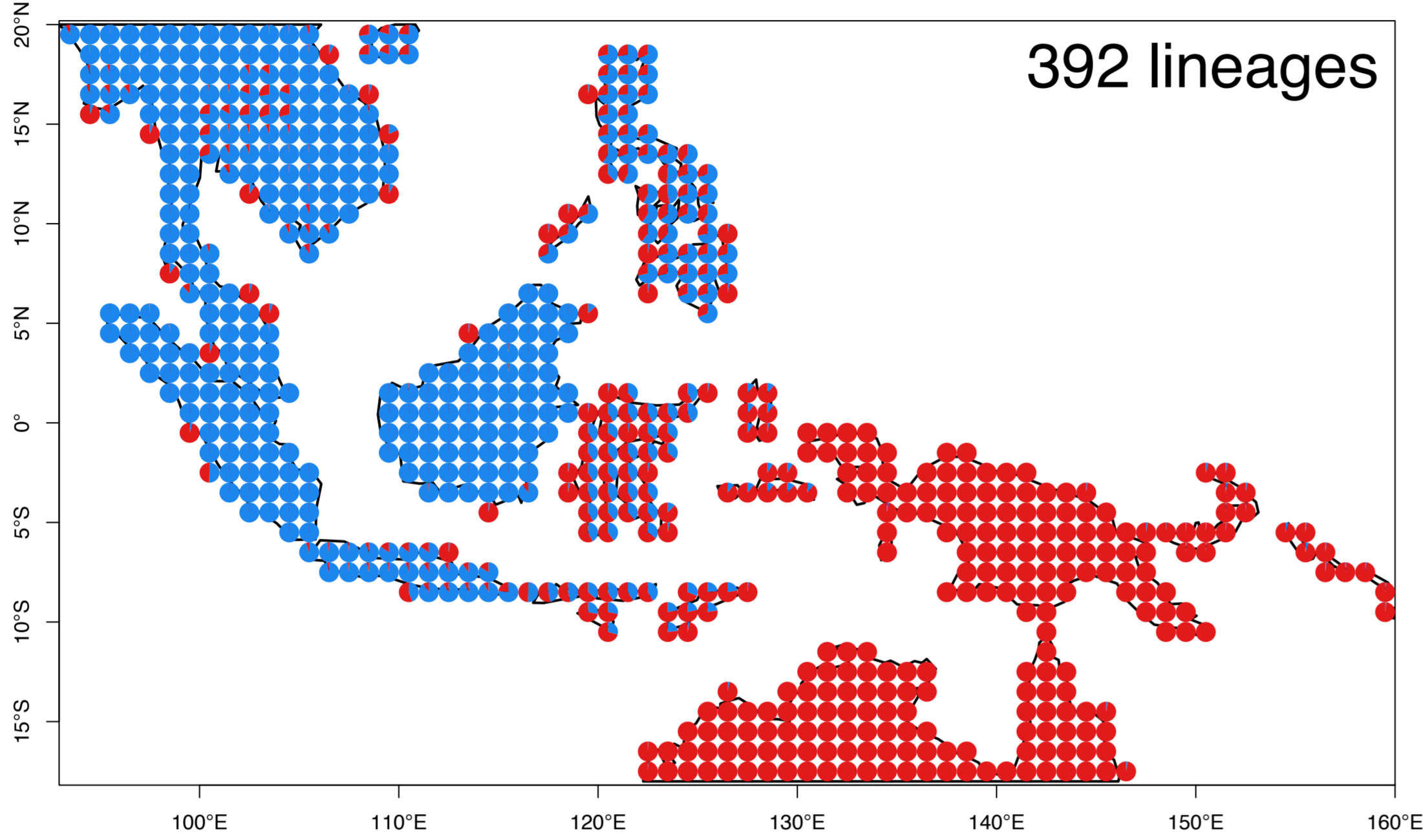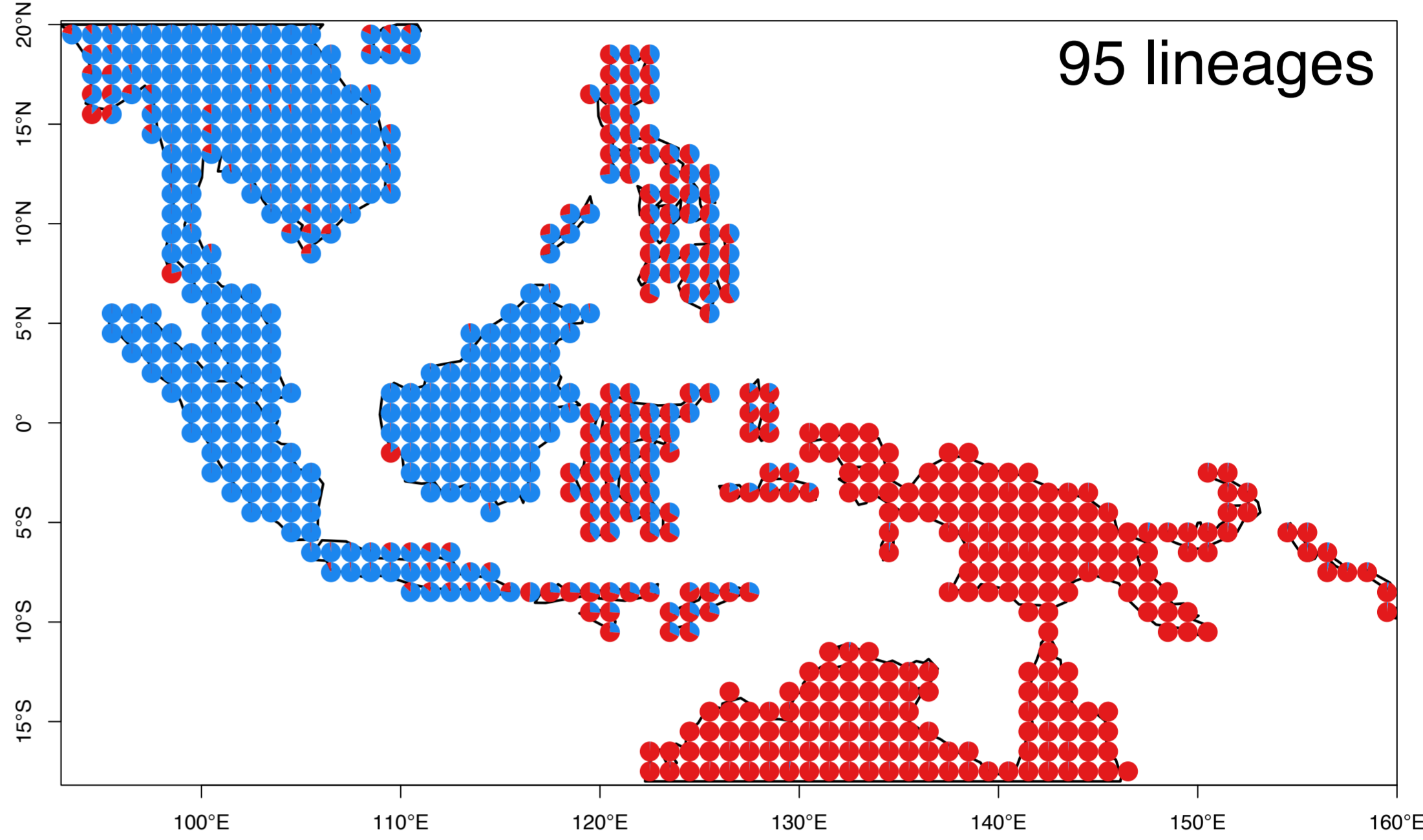

30 ma

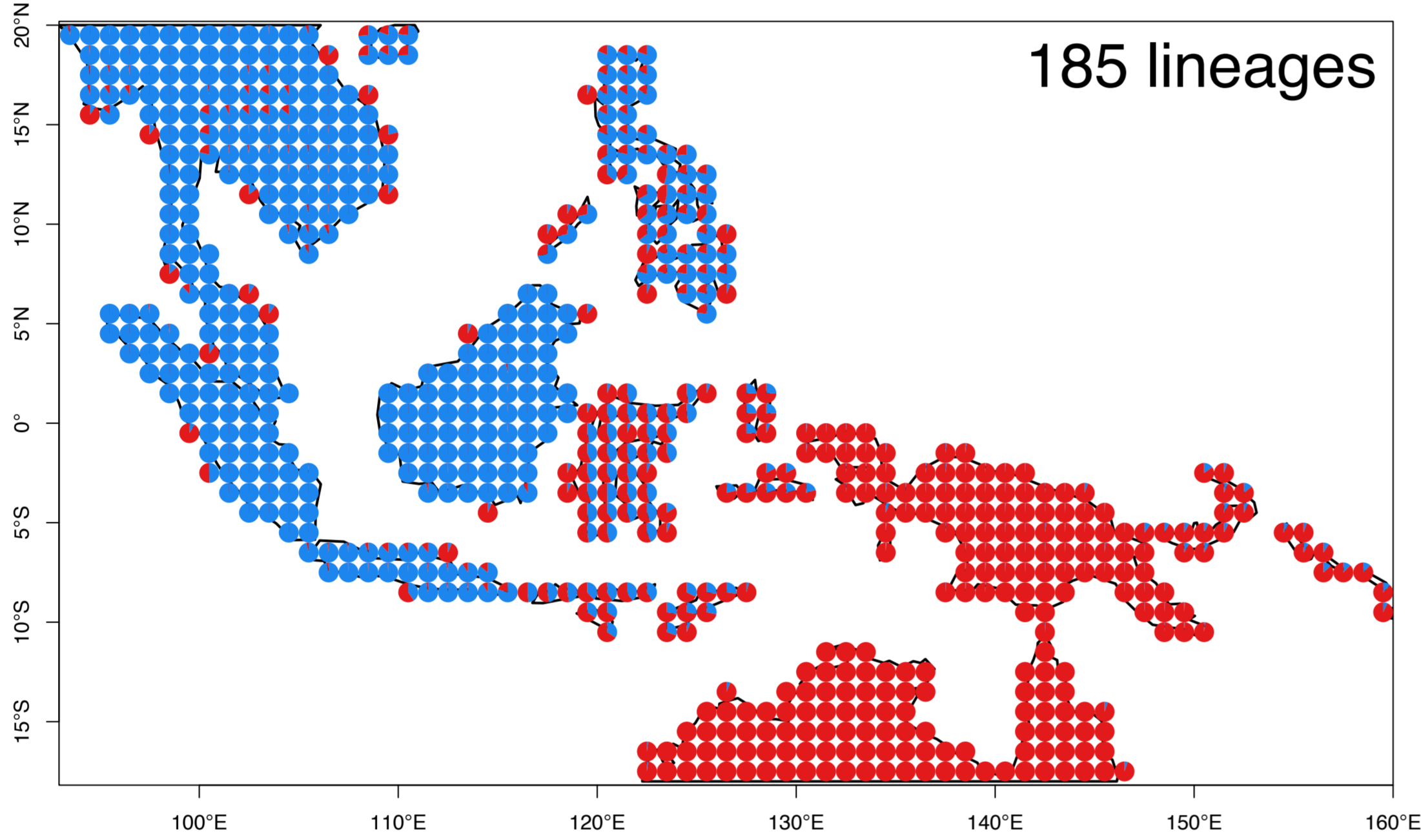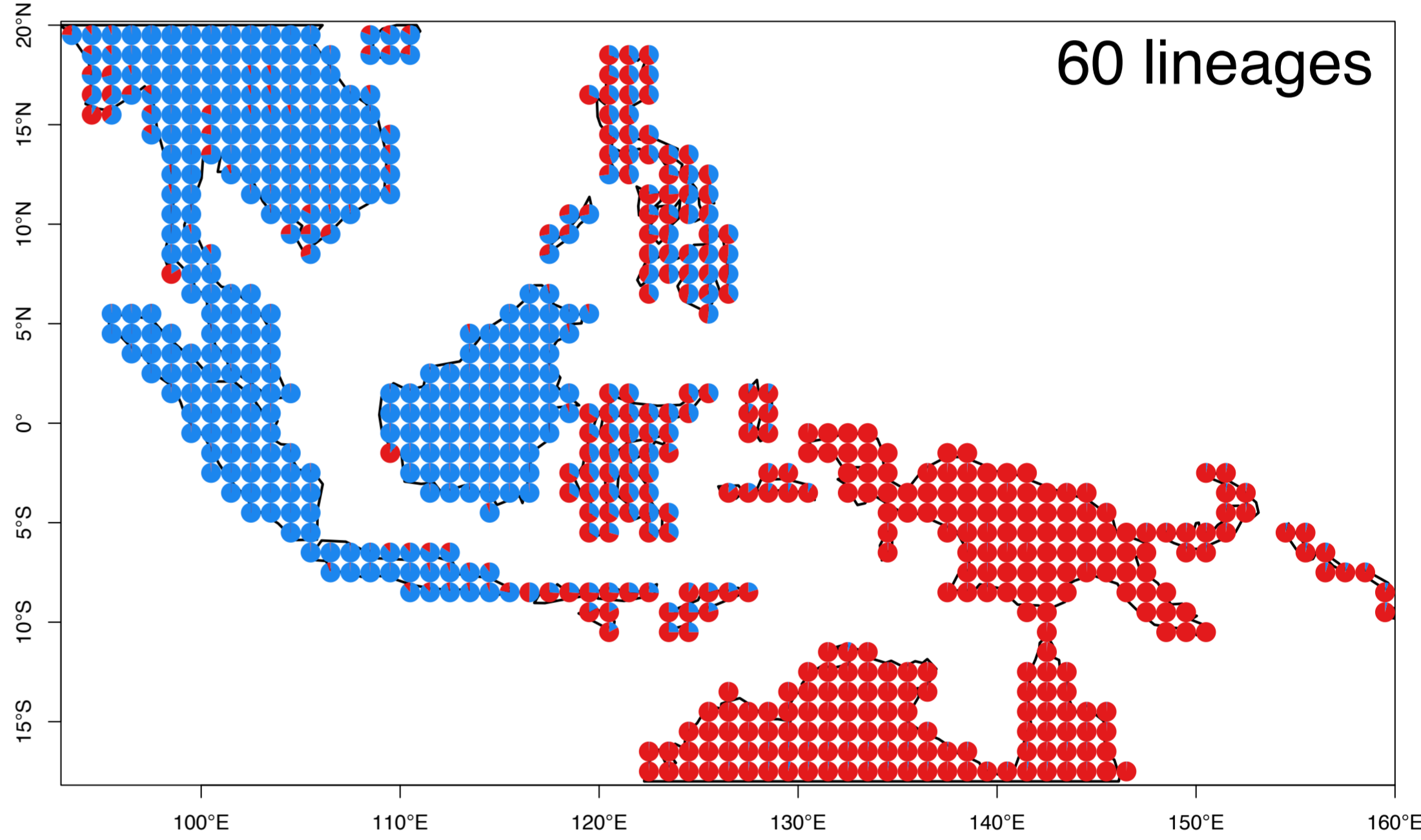

40 ma

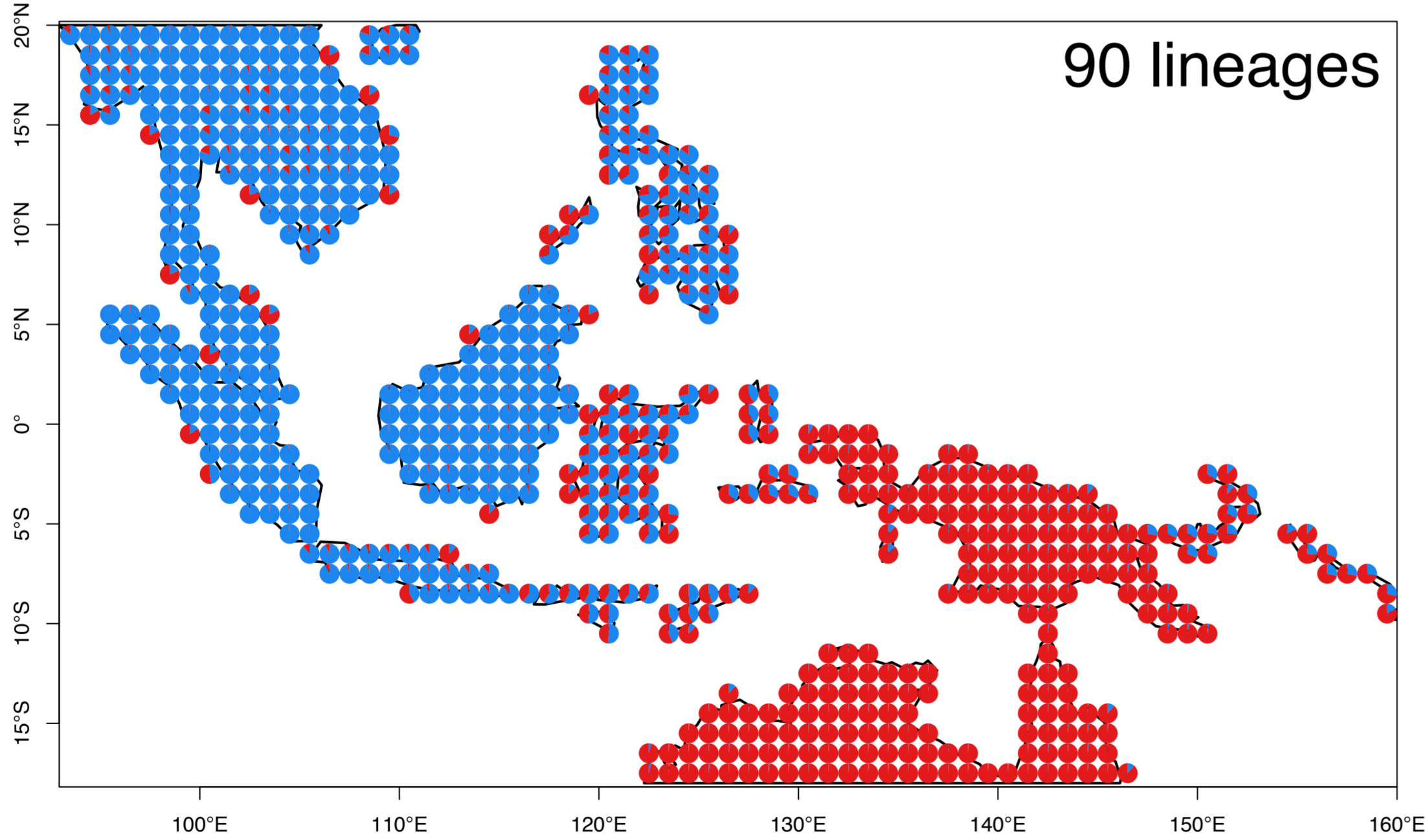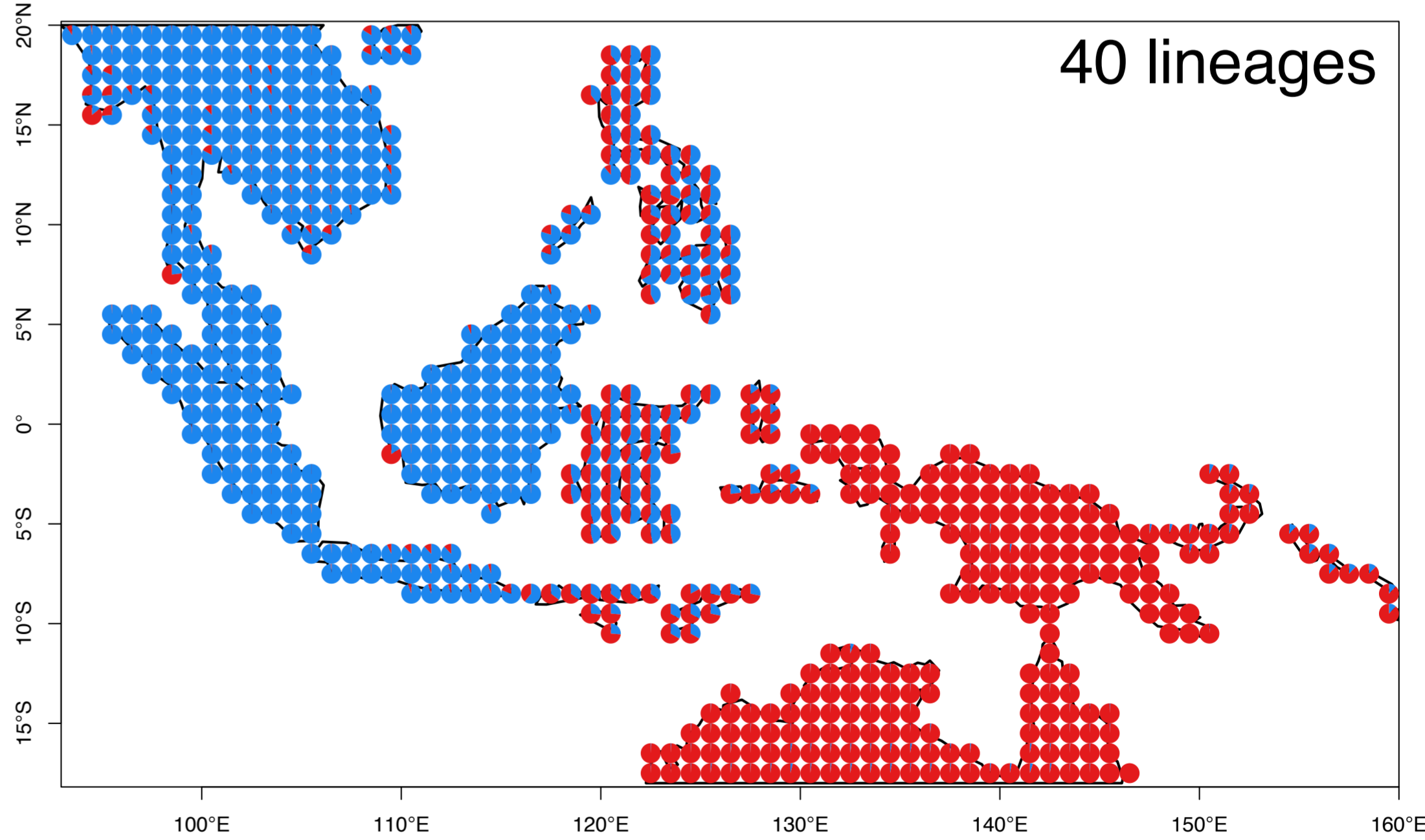

50 ma

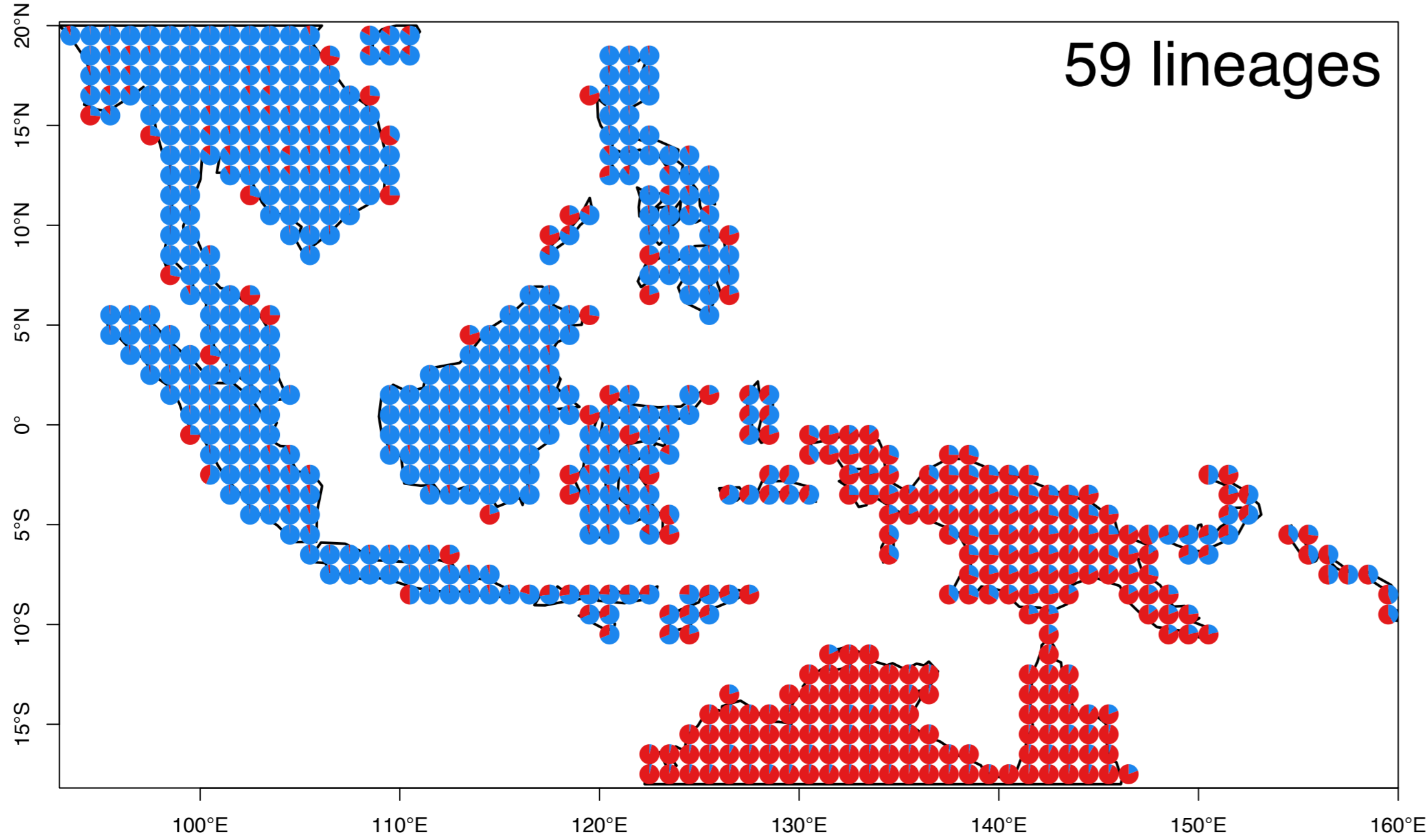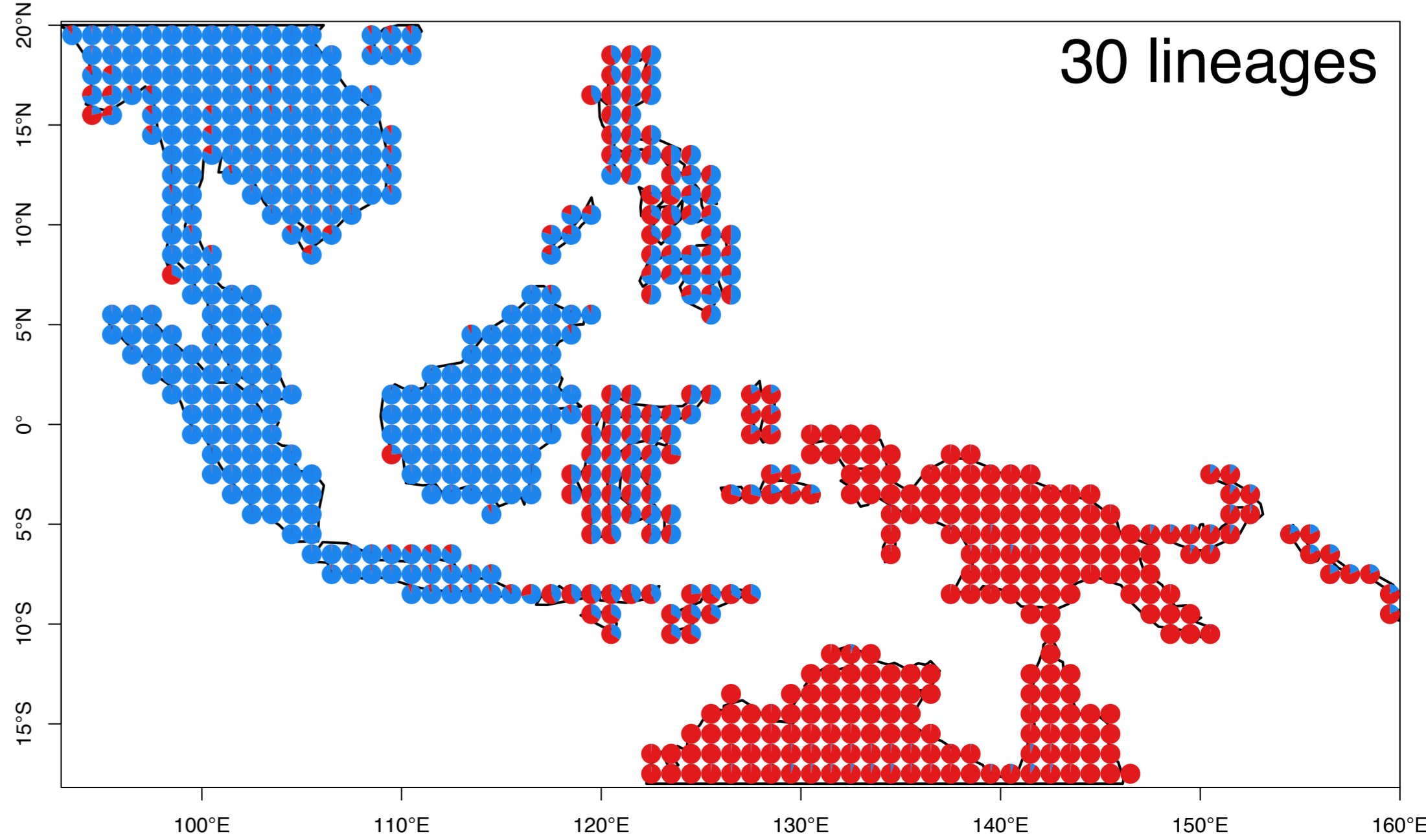

Supplement: Supplementary file 4 — Fig S4 [file GEB-30-685-s006.pdf]

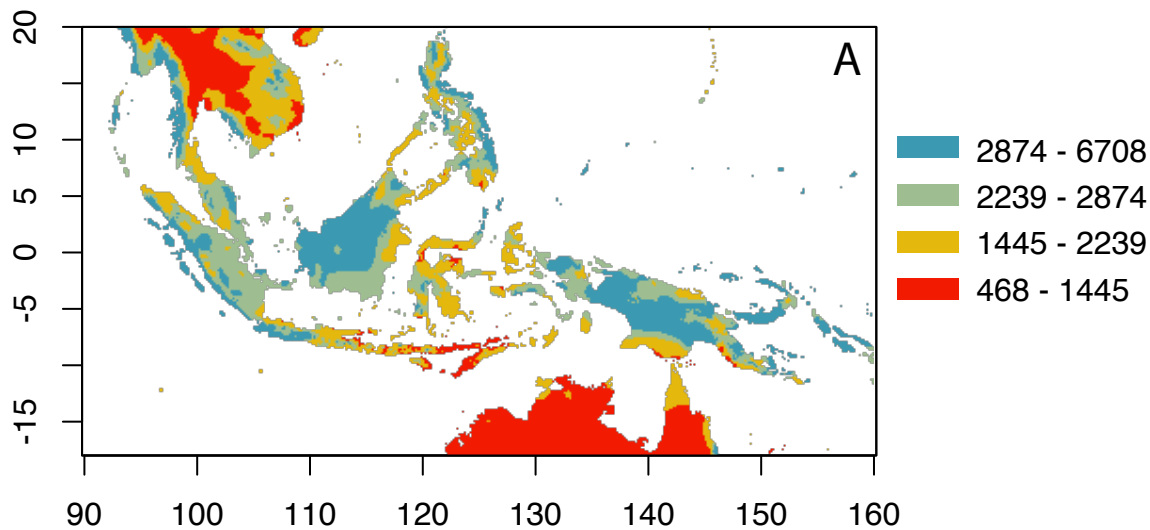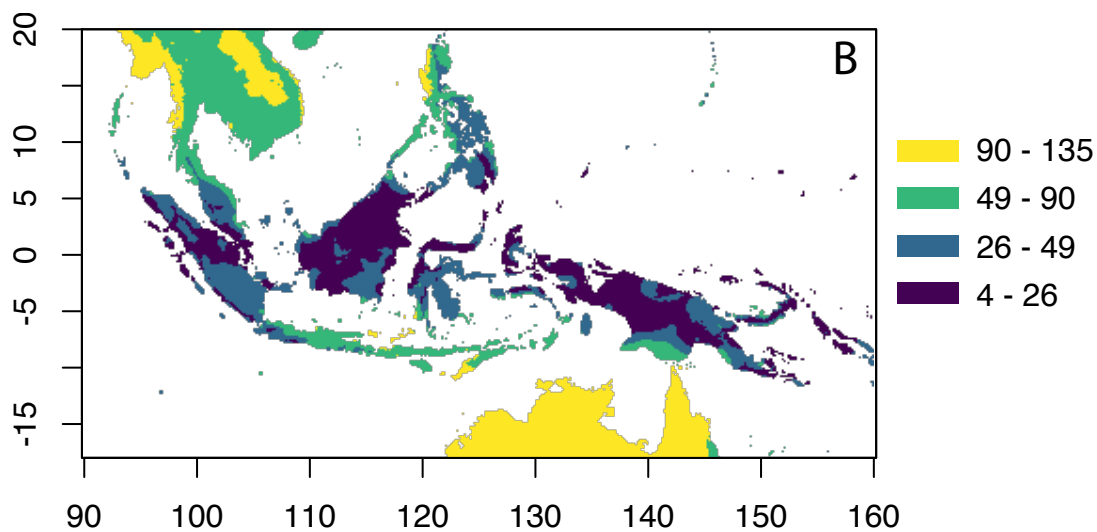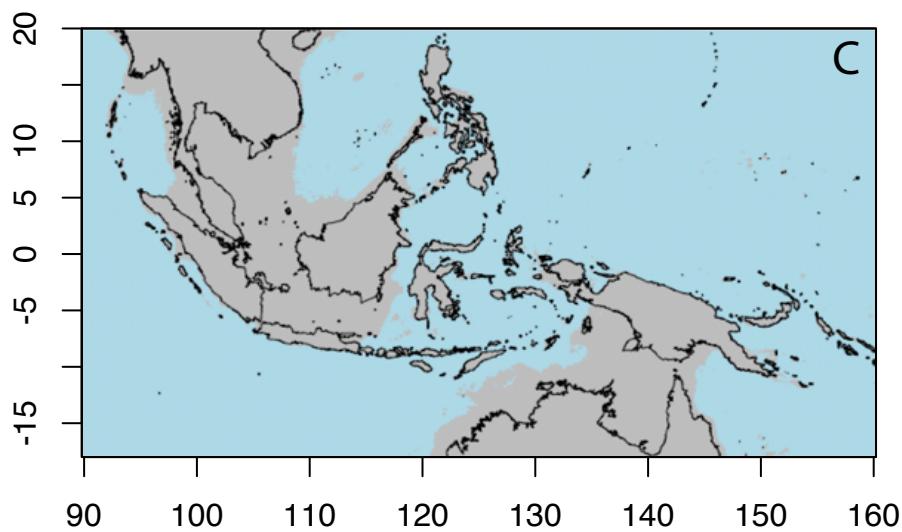

Supplement: Supplementary file 5 — Fig S5 [file GEB-30-685-s002.pdf]
