# Supplementary material for: Dispersal syndromes drive the formation of biogeographical regions, illustrated by the case of Wallace’s Line
Source: Glob Ecol Biogeogr. 2021 Jan 6;30(3):685–96. doi: 10.1111/geb.13250 (PMC7986858; doi:10.1111/geb.13250)
Supplement: Supplementary file 6 — Supplementary Material [file GEB-30-685-s001.docx]

| **Table S1.** Bird species widespread across Wallacea and found in Sulawesi, but which do  not extend west beyond the original Wallace-Huxley line |
| --- |
| *Aerodramus infuscatus* |
| *Aerodramus vanikorensis* |
| *Anas gracilis* |
| *Chalcophaps stephani* |
| *Circus assimilis* |
| *Coracina tenuirostris* |
| *Dendrocygna guttata* |
| *Egretta picata* |
| *Erythrura trichroa* |
| *Eudynamys orientalis* |
| *Eumyias panayensis* |
| *Hypotaenidia torquata* |
| *Lonchura molucca* |
| *Macropygia amboinensis* |
| *Megalurus timoriensis* |
| *Microcarbo melanoleucos* |
| *Monarcha cinerascens* |
| *Nectarinia aspasia* |
| *Pachycephala pectoralis* |
| *Pitta erythrogaster* |
| *Scythrops novaehollandiae* |
| *Tanygnathus sumatranus* |
| *Thalasseus bengalensis* |
| *Turnix maculosus* |

**Figure S1.** Map of the region with major islands marked for reference. Wallace’s original line is drawn.

**Figure S2.** Species motifs for birds and mammals in the Indo-Pacific, *K* = 7…9. Each pie chart is coloured according to contributions to the location from the inferred biotas.

**Figure S3.** *K*=10. Motifs for birds and mammals in the Indo-Pacific at three time slices, 0 (**A,B**), 5 Ma (**C,D**), and 50 Ma (**E,F**). Each pie chart is coloured according to contributions to the location from the 10 regional biotas.

**Figure S4.** Maps of phylogenetic motifs for K = 2. Note how Sulawesi and the Philippines become progressively more Asian at deeper levels in the tree. Figure 3 in the main text gives proportional make up through time for these two locations.

**Figure S5.** Precipitation (A, mm) and precipitation variability (B, coefficient of variation) across Wallacea and the Sunda and Sahul shelves (Bioclim 12 and 15 from Worldclim). Data are divided into quartiles and colours represent each quartile. C) Land area at the time of the last glacial maximum, 18,000 years ago (from E. J. Sbrocco and P. H. Barber. (2013). MARSPEC: ocean climate layers for marine spatial ecology. *Ecology* 94, 979, www.marspec.org)
